# Bridging Body Image Disturbance and Body Dysmorphic Disorder Symptoms: A Symptom Network Study Among University Students

**DOI:** 10.1002/brb3.71538

**Published:** 2026-06-08

**Authors:** Mohammed A. Mamun, S. M. Rakibul Hasan, Moneerah Mohammad ALmerab, Firoj Al‐Mamun

**Affiliations:** ^1^ CHINTA Research Bangladesh Dhaka Bangladesh; ^2^ Department of Public Health University of South Asia Dhaka Bangladesh; ^3^ Department of Psychology, College of Education and Human Development Princess Nourah Bint Abdulrahman University Riyadh Saudi Arabia

**Keywords:** body dysmorphic disorder, body image disturbance, bridge symptoms, network analysis, symptom network

## Abstract

**Background:**

Body image disturbance (BID) and body dysmorphic disorder (BDD) are common among university students and may impair emotional, social, and academic functioning. However, symptom‐level links between BID and body dysmorphic symptoms remain unclear, particularly in non‐Western student populations. This study used network analysis to examine central and bridge symptoms connecting BID and BDD symptoms among Bangladeshi university students.

**Methods:**

A cross‐sectional survey was conducted among 1401 university students in Bangladesh. Participants completed the Body Image Disturbance Questionnaire (BIDQ; seven items) and the Body Dysmorphic Disorder Screener for DSM‐5 (BDDS‐5; 11 core symptom items). A Gaussian graphical model was estimated using EBICglasso with an ordinal correlation matrix computed using *cor_auto*. Node centrality, bridge centrality, and predictability were examined. Network stability was assessed using bootstrapping, and gender differences were evaluated using the Network Comparison Test.

**Results:**

The estimated network showed distinct but interconnected BIDQ and BDDS‐5 symptom communities. The most central symptoms were comparing appearance with others, academic/professional impact, and feeling bad or miserable about appearance. Overall, the strongest bridge symptoms were academic/professional impact (bridge strength = 0.757), concern about a specific appearance feature (0.577), and interference with social life (0.442). The network showed acceptable stability (CS coefficient for node strength = 0.52). Global network strength did not differ significantly between males and females (7.86 vs. 8.13; *p* = 0.140), but network structure differed significantly (*M* = 0.230, *p* = 0.041). Among males, the strongest bridge symptoms were social/occupational impairment, academic/professional impact, and specific appearance concern; whereas specific appearance concern, social interference, trouble focusing due to appearance, and appearance‐related emotional distress were for females.

**Conclusion:**

This symptom‐level network analysis highlights academic/professional impairment, specific appearance concern, and social interference as key links between BID and BDD symptoms. Findings may inform targeted, gender‐sensitive interventions for university students and extend body image network research to a large Bangladeshi sample.

## Graphical abstract

This network analysis of Bangladeshi university students identifies academic/professional impact, specific appearance concern, and social interference as key bridge symptoms linking body image disturbance and body dysmorphic symptoms, with gender‐specific differences in symptom pathways that may inform targeted prevention and intervention strategies.

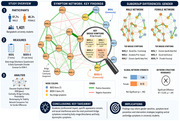

## 1 Introduction

Body image disturbance (BID) is a prominent psychological issue among adolescents and young adults, frequently co‐occurring with body dysmorphic disorder (BDD) and contributing to considerable distress and impaired daily functioning. BID is characterized by persistent dissatisfaction or negative perceptions of one's body size, shape, or specific features, often leading to emotional discomfort, social withdrawal, and repeated attempts to alter appearance (Frederick et al. 2012). Such concerns are pervasive: for example, 78.8% of Pakistani medical students reported dissatisfaction with some aspect of their appearance (Taqui et al. 2008), while studies from Ethiopia (Jebero et al. 2025) and India (Mohanty et al. 2024) also document high rates of body‐related distress, avoidance, and academic interference. Both males and females are affected, although the focus of concern may differ, with females more often reporting preoccupation with weight or shape and males more often reporting concerns related to muscularity and leanness; these concerns are influenced by factors such as media exposure, family attitudes, nutrition, and peer pressure (Hoffmann et al. 2018; Kapoor et al. 2022). In South Asian contexts, body image concerns may be further shaped by culturally specific appearance ideals, including pressures related to skin tone, facial features, body shape, and socially valued standards of attractiveness, alongside strong family‐ and peer‐based scrutiny of appearance and social presentation (Chan and Hurst 2022; Mishra et al. 2023; Rodgers et al. 2023). Among university students, these pressures may intersect with emerging adulthood, identity formation, interpersonal expectations, and academic competition, thereby increasing vulnerability to both general BID and more severe dysmorphic symptoms (Mishra et al. 2023; Rodgers et al. 2023).

BDD is a more severe and specific psychiatric condition marked by persistent, intrusive thoughts about perceived physical flaws, usually minor or unnoticeable to others (Mufaddel et al. 2013). Individuals with BDD may engage in repetitive behaviors such as mirror checking, reassurance seeking, excessive grooming, skin picking, or frequent appearance comparisons in attempts to reduce their distress (American Psychiatric Association 2022). These symptoms can result in significant embarrassment, social isolation, and disruptions in academic or occupational functioning. Reflecting its overlap with obsessive‐compulsive features, the DSM‐5 classifies BDD under obsessive‐compulsive and related disorders (American Psychiatric Association 2022). Prevalence estimates suggest that BDD affects 0.7%–2.4% of the general population, with higher rates among psychiatric inpatients, cosmetic or dermatological patients, and university students, particularly women (McGrath et al. 2023; Mohanty et al. 2024; Mufaddel et al. 2013; Saab et al. 2023; Taqui et al. 2008). The disorder often emerges in adolescence or young adulthood and is frequently comorbid with depression and anxiety, contributing to substantial psychological and functional burden. Although BID and BDD are conceptually distinct, they are closely related and may overlap at the symptom level, making it important to examine how broader body image concerns connect with more severe dysmorphic symptoms (Schmidt et al. 2022).

Recently, psychological network analysis has emerged as a useful method for mapping the complex interconnections among mental health symptoms (Borsboom 2017), enabling the identification of central and bridge features within conditions such as BID and BDD. Within this framework, symptoms are not viewed merely as passive indicators of an underlying disorder; rather, they are conceptualized as interacting components that may reinforce one another over time (Borsboom 2017; Robinaugh et al. 2020). This framework is especially relevant for body‐image‐related psychopathology, in which distress, avoidance, preoccupation, reassurance seeking, and functional impairment may form mutually reinforcing feedback loops. However, previous network studies have differed widely in their analytic focus, sample characteristics, and, importantly, the assessment tools used for body image dissatisfaction and BDD. For example, Fuller‐Tyszkiewicz et al. (2020) conducted an item‐level network analysis of body dissatisfaction—covering both specific body‐part concerns and body functions—using the Body Dissatisfaction subscale of the Body Image in Pregnancy Scale among pregnant and non‐pregnant Australian women. They found that shape and weight concerns were most central, but did not assess BDD or use functionally oriented measures such as the Body Image Disturbance Questionnaire (BIDQ). In contrast, Summers et al. (2020) applied network analysis to the BDD–Yale‐Brown Obsessive Compulsive Scale (BDD‐YBOCS) and a depression scale in a clinical sample of 148 BDD patients in the United States, identifying compulsive behaviors, interference in daily life, and feelings of worthlessness as central symptoms. However, this study did not address broader body image dissatisfaction or utilize the BIDQ or Body Dysmorphic Disorder Screener for DSM‐5 (BDDS‐5), thus leaving out the full spectrum from general dissatisfaction to clinical dysmorphia. Another study of individuals with gender dysphoria used the Body Image Scale for Transsexuals to examine satisfaction with various body features in a multi‐country European sample, providing insight into body satisfaction patterns but not directly addressing BDD or general BID as primary constructs (Van de Grift et al. 2016).

Other studies have explored related psychopathology using a range of measurement tools and analytic strategies, but with differing emphases on BID or BDD. For example, Finch et al. (2023) investigated comorbidity between eating disorders and premenstrual symptoms in a sample of young Australian women by constructing a symptom network using the Eating Pathology Symptoms Inventory and the Daily Record of Severity of Problems. While their analysis identified central and bridge symptoms connecting eating pathology and premenstrual complaints, it did not directly assess body image dissatisfaction or BDD as standalone constructs. Similarly, a study of Italian adults modeled the interplay among subscales of the Eating Disorder Inventory‐3, the Body Appreciation Scale, and the Intuitive Eating Scale to examine protective and risk factors for eating disorders. Although body dissatisfaction was included, neither BDD nor the BIDQ or BDDS‐5 were assessed (Cerea et al. 2024). In India, item‐level network analyses of interoceptive sensibility and self‐objectification were conducted among women with high and low levels of body dissatisfaction, focusing on dissatisfaction network structure but not on BDD or clinical screening tools (Naraindas et al. 2025). Dingemans et al. (2022) further expanded network approaches to the obsessive‐compulsive spectrum, analyzing networks including BDD, eating disorders, OCD, and autism spectrum symptoms in Dutch and German clinical and control groups using multiple disorder‐specific scales; however, BID was not a primary focus, nor were the BIDQ or BDDS‐5 used. This measurement heterogeneity limits direct comparison across studies and makes it difficult to determine whether the most influential symptoms reflect broader body dissatisfaction, clinically relevant dysmorphic concerns, or both.

Although prior network analyses have provided important insights into body dissatisfaction, BDD, and related psychopathology, to our knowledge, no previous study has jointly modeled item‐level associations between the BIDQ and the BDDS‐5, particularly among university students. This gap is important because university students represent a developmentally and socially salient group in whom appearance‐related concerns may have direct implications for emotional well‐being, peer relationships, and academic functioning. To address this gap, the present study applied network analysis to a large, gender‐balanced sample of Bangladeshi university students (*N* = 1401), modeling BIDQ and BDDS‐5 items as network nodes. The primary aims were to: (i) identify central symptoms within the combined BID and BDD network, (ii) detect bridge symptoms linking the two symptom domains, and (iii) examine subgroup differences by gender. Based on prior work on body‐image‐related psychopathology, we expected that symptoms reflecting functional impairment, social interference, and appearance‐related preoccupation would emerge as especially influential within the network and may serve as bridges linking broader BID with dysmorphic symptoms (Chan and Hurst 2022; Mishra et al. 2023; Rodgers et al. 2023). This approach may clarify the interrelations between BID and dysmorphic symptoms in a culturally distinct, under‐researched population and inform future targeted prevention and intervention efforts.

## 2 Methods

### 2.1 Study Design, Site, and Participants

This study was conducted at Jahangirnagar University, Savar, Dhaka, Bangladesh, from December 18, 2024 to January 10, 2025. Participants were recruited from among students residing in university dormitories during the study period. To be included in the study, participants were required to (i) reside in university dormitories, (ii) be at least 18 years old, and (iii) be willing to participate and provide consent for the study. The research team approached students in their dormitories, explained the study's aims and procedures, and invited them to participate. Written informed consent was obtained from each participant prior to data collection. Data were collected using a self‐administered, structured questionnaire, which required approximately 30 min to complete. A total of 1447 students were surveyed using a convenience sampling approach. Respondents who were older than 29 years or who failed to answer most of the key outcome variables were excluded from the final analysis. As a result, the final dataset comprised 1401 students, yielding a valid completion rate of 96.8%. Because participants were recruited from university dormitories using convenience sampling, the sample may overrepresent students with high levels of peer interaction and shared campus‐related social experiences, which should be considered when interpreting the generalizability of the findings.

### 2.2 Measures

#### 2.2.1 Body Image Disturbance Questionnaire

This study employed the BIDQ, a 7‐item measure developed to assess body image concerns and their effects on daily functioning (Cash et al. 2004). Each item is scored on a 5‐point Likert scale ranging from 1 (“*not at all concerned*”) to 5 (“*extremely concerned*”), yielding a total score range of 7–35. The BIDQ covers several dimensions of BID, including concern about unattractive body parts, preoccupation with these concerns, emotional distress, impairment in social or occupational functioning, interference with social life, impact on academic or role‐related activities, and avoidance behaviors related to body dissatisfaction. The original BIDQ showed high internal consistency (*α* = 0.89) (Cash et al. 2004). In the present study, the BIDQ also demonstrated excellent reliability (*α* = 0.90), supporting its adequacy for use in this Bangladeshi university student sample.

#### 2.2.2 Body Dysmorphic Disorder Screener for DSM‐5

This study employed the BDDS‐5, a 12‐item screening instrument developed to assess body dysmorphic symptoms in accordance with DSM‐5 criteria (Van Rood et al. 2023). The measure includes items covering appearance‐related preoccupation, repetitive behaviors, distress, and functional impairment, as well as an exclusion item assessing whether the concerns are better explained by weight‐ or eating‐related problems. Each item uses dichotomous response options (“True/Yes” and “False/Not true/No”). In the present study, BDDS‐5 item‐level responses were used for symptom network analysis rather than categorical screening classification. For the primary network model, 11 core BDDS‐5 symptom items (BDDS_1–BDDS_11) were included alongside the seven BIDQ items. BDDS_12 was excluded from the primary network because it reflects an exclusion criterion related to weight‐ and eating‐disorder concerns rather than a core symptom of BDD. To assess the robustness of the findings, a sensitivity analysis was subsequently conducted in which BDDS_12 was also included in an alternative network specification.

### 2.3 Ethics Statement

The authors affirm that all procedures contributing to this research adhered to the ethical standards of the relevant national and institutional committees governing human research, as well as the principles outlined in the Declaration of Helsinki (1975, revised 2013). Ethical approval for all study procedures involving human participants was obtained from the ethics committee at CHINTA Research Bangladesh (Reference: CHINTA/IRB/10‐2024/17). Before initiating the survey, participants were fully informed about the study's objectives, procedures, potential risks, and their rights—including the right to withdraw from the study at any time without penalty. Written informed consent was obtained from all participants to confirm their understanding and voluntary participation. To protect confidentiality, all data were securely stored and accessible only to the research team, and were used solely for research purposes. No monetary or non‐monetary incentives were provided for participation.

### 2.4 Statistical Analysis

#### 2.4.1 Item Selection and Data Preparation

For the primary network analysis, we focused on item‐level responses from the BIDQ (7 items) and 11 core symptom items from the BDDS‐5, yielding a total of 18 symptom nodes. The BDDS‐5 exclusion item related to weight‐ and eating‐disorder concerns (BDDS_12) was not included in the primary network because it reflects a diagnostic exclusion criterion rather than a core body dysmorphic symptom. As the final analytic dataset contained no missing values for the selected items, all 1,401 participants were retained in the network analyses.

#### 2.4.2 Network Estimation

The estimated network represented conditional associations among the seven BIDQ items and 11 core BDDS‐5 symptom items. We estimated a Gaussian graphical model (GGM) using the Extended Bayesian Information Criterion Graphical Least Absolute Shrinkage and Selection Operator (EBICglasso) method, implemented in the *bootnet* package (Epskamp et al. 2018). Given the ordered categorical BIDQ responses and dichotomous BDDS‐5 responses, the network was estimated using an ordinal correlation matrix computed through the cor_auto function. The resulting weighted adjacency matrix represented regularized partial correlations between nodes, controlling for all other nodes in the network. Diagonal elements were set to zero to remove self‐loops.

#### 2.4.3 Network Visualization

The estimated network was visualized using the *qgraph* package (Epskamp et al. 2012). Nodes were color‐coded by scale (BIDQ and BDDS‐5), and the highest‐ranking bridge symptoms were highlighted in the network visualization to facilitate interpretation. Node labels reflected item content for interpretability. A heatmap of edge weights was also generated to illustrate the relative strength and direction of inter‐item associations.

#### 2.4.4 Centrality and Predictability

Node centrality metrics, including strength and expected influence (1‐step and 2‐step), were calculated to quantify the relative importance of each symptom within the network. Strength was computed as the sum of the absolute edge weights connected to each node, while expected influence was calculated as the sum of signed edge weights. Because the network contained some negative edges, expected influence was examined alongside strength to aid the interpretation of node importance. In addition, a predictability proxy (*R* ^2^) was computed for each node by summing the squared non‐zero edge weights connected to that node, providing an approximate index of local connectivity rather than a formal model‐based estimate of explained variance. Centrality indices and the predictability proxy were visualized using four‐panel plots.

#### 2.4.5 Bridge Centrality

To identify potential symptoms that statistically connected BID and BDD symptom domains, we conducted bridge centrality analysis using the *networktools* package (Jones et al. 2021). Community assignments were defined as BIDQ and BDDS‐5 item sets, and bridge centrality indices, including bridge strength and bridge expected influence (1‐step and 2‐step), were computed. These two communities represented broader BID and body dysmorphic symptom domains, respectively. The top bridge nodes were visualized and tabulated for interpretation.

#### 2.4.6 Network Stability and Accuracy

We assessed the accuracy and stability of the estimated network using nonparametric bootstrapping (2000 samples) as implemented in the bootnet package. Edge‐weight accuracy was evaluated by constructing 95% bootstrap confidence intervals around the estimated edge weights. Centrality stability (CS) was quantified using the case‐dropping subset bootstrap, resulting in a CS coefficient; values above 0.25 were considered acceptable, and above 0.50 were considered good (Epskamp et al. 2018).

#### 2.4.7 Network Comparison Test

To examine gender differences in network structure, we conducted a Network Comparison Test (NCT) using the *NetworkComparisonTest* package (van Borkulo et al. 2022) with 1000 permutations. Separate Gaussian graphical models were estimated for males and females using the same analytic procedures as the total sample. The NCT evaluated global strength invariance, network structure invariance (*M* statistic), edge‐specific differences, and centrality invariance for node strength. Bridge centrality indices were also computed for each gender‐specific network to identify and compare bridge symptoms across groups. All statistical outputs, including observed differences, permutation‐based *p*‐values, significant edge or centrality differences, and gender‐specific bridge centrality values, are reported in Section 3 and Supporting Information tables. Gender‐specific networks were visualized side‐by‐side using a common node layout, with the top three bridge symptoms in each group highlighted for interpretability.

#### 2.4.8 Sensitivity Analysis

A sensitivity analysis was conducted using an alternative network specification in which BDDS_12, the BDDS‐5 exclusion item related to weight‐ and eating‐disorder concerns, was additionally included. This analysis was conducted to examine whether the main pattern of results was robust to the inclusion of this exclusion item. Centrality, bridge centrality, and gender‐based network comparison results from the sensitivity analysis were compared with those from the primary network model.

#### 2.4.9 Software

All analyses were conducted in R version 4.4.1, primarily utilizing the *bootnet* (v1.5.1), *qgraph* (v1.9), *networktools* (v1.4.0), and *NetworkComparisonTest* (v2.2.1) packages. Detailed Supporting Information tables and figures are provided to support the interpretation of the analyses.

## 3 Results

### 3.1 Descriptions of the Study Participants

A total of 1401 university students participated in the study, with 51.7% male and a mean age of 22.96 years (SD = 2.01). Among participants, 16.6% were classified as overweight and 3.4% as obese. Current tobacco use was reported by 19.4%, and 51.2% did not engage in regular physical activity. The most commonly reported appearance‐related concerns were hair (32.0%), acne (29.1%), and scar marks (17.8%), followed by skin tone (10.4%), teeth (9.6%), and weight (2.5%). The mean score on the BIDQ was 12.0 (SD = 5.39), and the mean score on the BDDS‐5 was 3.37 (SD = 2.76). Gender‐based analyses indicated that female students scored significantly above males on both scales, including the BIDQ (12.70 ± 5.60 vs. 11.33 ± 5.09, *p* < 0.001) and the BDDS‐5 (3.53 ± 2.92 vs. 3.22 ± 2.60, *p* = 0.036). Participant characteristics are summarized in Table 1.

**TABLE 1:** Description of the study participants.

Variables	Total Sample (*n*, %)
Gender	
Male	722, 52%
Female	675, 48%
Body mass index	
Underweight	137, 9.8%
Normal	983, 70%
Overweight	233, 17%
Obese	48, 3.4%
Current smoking status	
No	1118, 79.8%
Yes	263, 19.2%
Current physical activities	
No	714, 51%
Yes	680, 48.5%
Discomfort with specific body features	
Acne	408, 29.1%
Skin tone	146, 10.4%
Tooth	134, 9.6%
Scar marks	249, 17.8%
Hair	449, 32%
Weight	35, 2.5%
Others^a^	22, 1.6%

^a^ Other**s**: height, throat, facial hair, hair fall, and nose.

### 3.2 Network Estimation and Structure

**FIGURE 1:**
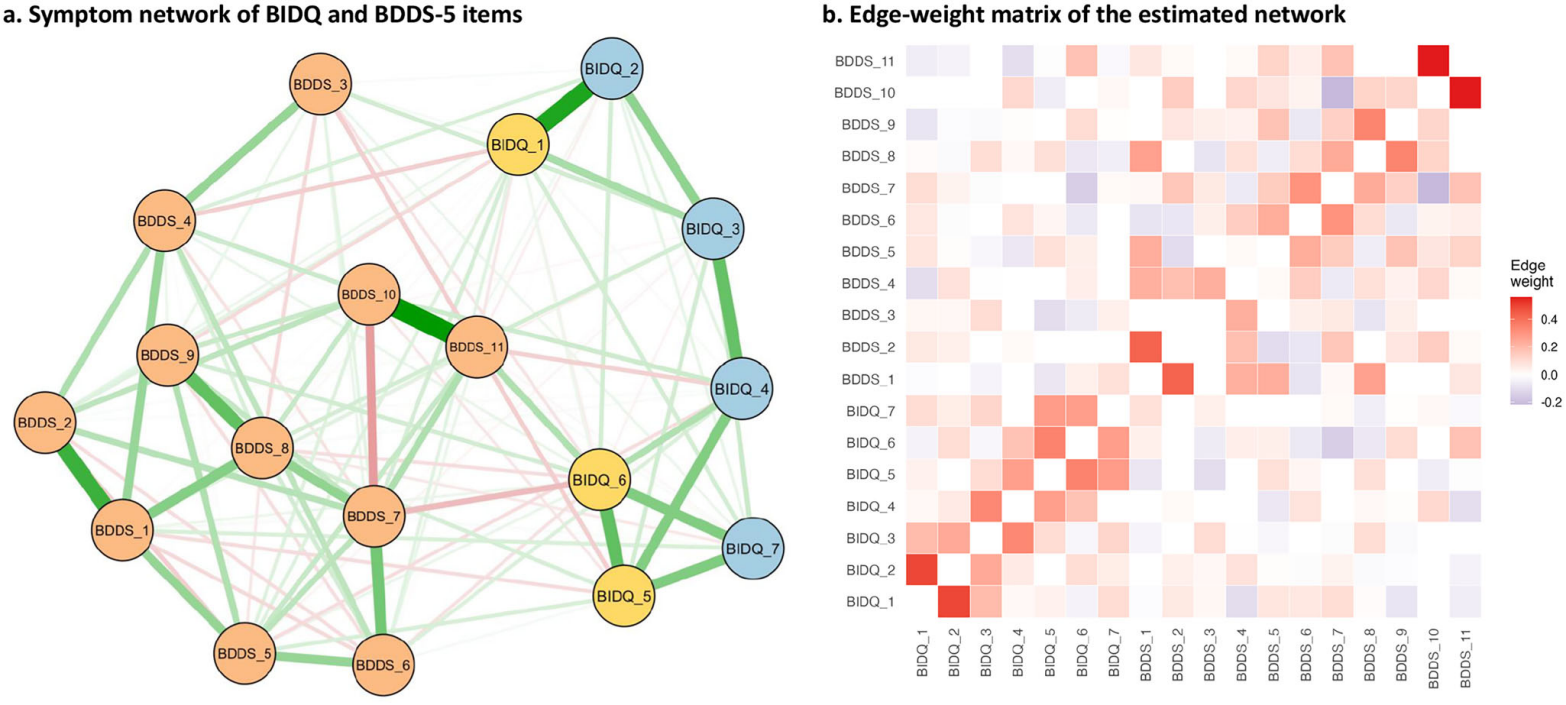
Estimated symptom network and regularized edge‐weight matrix. (a) Estimated symptom network of BIDQ and BDDS‐5 items. Blue nodes indicate BIDQ items, orange nodes indicate BDDS‐5 items, and yellow nodes indicate the highest‐ranking bridge nodes. Edge thickness represents the magnitude of regularized partial correlations; green edges denote positive associations and red edges denote negative associations. (b) Regularized edge‐weight matrix corresponding to the estimated network. Cell color represents the magnitude and direction of each partial correlation (red = positive; blue = negative).

The estimated symptom network comprised 18 items from the BIDQ (7 items) and the BDDS‐5 (11 core symptom items) and is shown in Figure 1a. Edges represent regularized partial correlations adjusted for all other symptoms in the network. Visual inspection showed strong within‐community clustering, with BIDQ symptoms grouping together and BDDS‐5 symptoms forming a distinct but interconnected cluster. A heatmap of the corresponding regularized edge‐weight matrix is shown in Figure 1b, and the full weighted adjacency matrix is provided in Table S1.

Within the BIDQ community, the strongest positive edge was observed between BIDQ_1 (“Concern about appearance of specific body part”) and BIDQ_2 (“Preoccupation with perceived defect”; edge weight = 0.494), followed by BIDQ_5 (“Interference with social life”) and BIDQ_6 (“Impact on academic/professional activities”; edge weight = 0.347), and BIDQ_3 (“Emotional distress due to appearance concern”) and BIDQ_4 (“Impairment in social/occupational functioning”; edge weight = 0.335). Other relatively strong within‐BIDQ edges included BIDQ_5–BIDQ_7 (0.281) and BIDQ_6–BIDQ_7 (0.276). Within the BDDS‐5 community, the strongest positive edge was observed between BDDS_10 (“Difficult doing things with others”) and BDDS_11 (“Trouble focusing due to appearance”; edge weight = 0.564), followed by BDDS_1 (“I look strange or ugly”) and BDDS_2 (“I constantly think about appearance”; edge weight = 0.430), and BDDS_8 (“Feel bad or miserable about appearance”) and BDDS_9 (“Avoid activities due to appearance”; edge weight = 0.345). Other notable within‐BDDS edges included BDDS_6–BDDS_7 (0.307), BDDS_1–BDDS_8 (0.267), and BDDS_7–BDDS_8 (0.239).

Several negative edges were also present in the network. The largest negative edge was observed between BDDS_7 (“Comparing appearance with others”) and BDDS_10 (“Difficult doing things with others”; edge weight = −0.216). BIDQ_6 (“Impact on academic/professional activities”) also showed negative edges with BDDS_7 (“Comparing appearance with others”; edge weight = ‐0.147) and BDDS_8 (“Feel bad or miserable about appearance”; edge weight = −0.073).

Cross‐community edges were generally weaker than the strongest within‐community associations. The strongest positive cross‐domain edge was observed between BIDQ_6 (“Impact on academic/professional activities”) and BDDS_11 (“Trouble focusing due to appearance”; edge weight = 0.173), followed by BIDQ_4 (“Impairment in social/occupational functioning”) and BDDS_10 (“Difficult doing things with others”; edge weight = 0.109), and BIDQ_3 (“Emotional distress due to appearance concern”) and BDDS_3 (“Others do not think anything is wrong”; edge weight = 0.100). The strongest negative cross‐domain edge was BIDQ_6–BDDS_7 (edge weight = −0.147).

### 3.3 Centrality and Predictability Metrics

**FIGURE 2:**
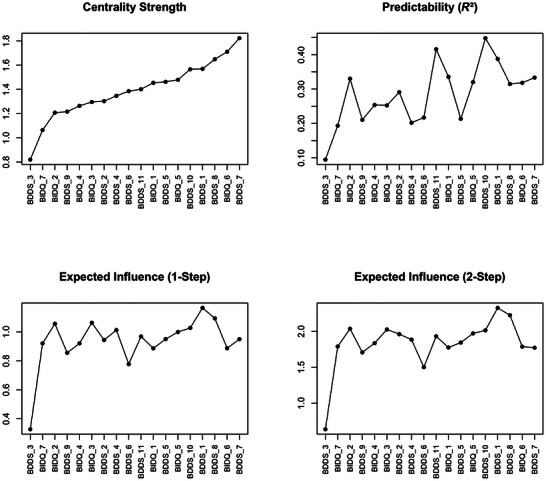
Centrality and predictability‐proxy estimates for the estimated BIDQ–BDDS‐5 symptom network. Panels display node strength, predictability proxy (*R* ^2^), one‐step expected influence, and two‐step expected influence. Nodes are ordered by strength.

Centrality indices were computed to quantify the relative importance of each symptom within the network (Figure 2). Based on strength, the most central nodes were BDDS_7 (“Comparing appearance with others”; strength = 1.822), BIDQ_6 (“Impact on academic/professional activities”; strength = 1.711), and BDDS_8 (“Feel bad or miserable about appearance”; strength = 1.649). High strength was also observed for BDDS_1 (“I look strange or ugly”; strength = 1.569) and BDDS_10 (“Difficult doing things with others”; strength = 1.566). Predictability values ranged from 0.095 for BDDS_3 to 0.448 for BDDS_10, indicating that some symptoms were only modestly explained by their directly connected neighboring nodes, whereas others showed moderate local interconnectedness. Because the network contained negative edges, expected influence was considered alongside strength; BDDS_1, BDDS_8, BIDQ_3, BIDQ_2, and BDDS_10 showed comparatively high one‐step expected influence values. Full centrality and predictability estimates are presented in Table S2.

### 3.4 Bridge Centrality

Bridge centrality analysis was conducted to identify symptoms connecting the BIDQ and BDDS‐5 communities. The highest bridge strength values in the overall network were observed for BIDQ_6 (“Impact on academic/professional activities”; bridge strength = 0.757), BIDQ_1 (“Concern about appearance of specific body part”; bridge strength = 0.577), BIDQ_5 (“Interference with social life”; bridge strength = 0.442), BIDQ_4 (“Impairment in social/occupational functioning”; bridge strength = 0.404), and BDDS_11 (“Trouble focusing due to appearance”; bridge strength = 0.390). Among the BDDS‐5 items, the highest bridge strength values were observed for BDDS_11, BDDS_8 (“Feel bad or miserable about appearance”; bridge strength = 0.357), BDDS_3 (“Others do not think anything is wrong”; bridge strength = 0.353), and BDDS_5 (“Skin picking/clothing adjustment”; bridge strength = 0.311). Detailed bridge centrality estimates are presented in Table S3.

### 3.5 Accuracy and Stability of the Network

**FIGURE 3:**
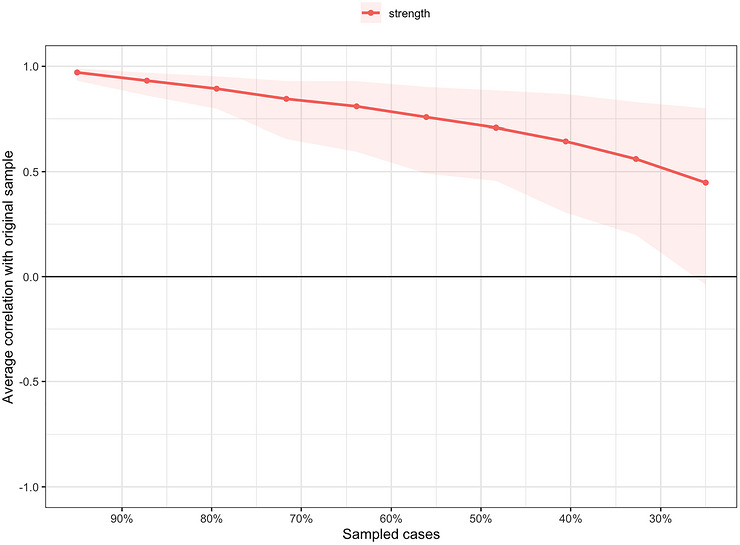
Case‐dropping bootstrap analysis of strength centrality stability in the BIDQ–BDDS‐5 symptom network. The red line shows the average correlation between centrality estimates from subsamples and the original sample across increasing proportions of dropped cases; shaded bands represent 95% confidence intervals.

The accuracy and stability of the estimated network were evaluated using nonparametric bootstrapping with 2000 resamples. Case‐dropping bootstrap analysis of node strength is shown in Figure 3. Additional bootstrap results are presented in Figure S1 (bootstrap edge‐weight confidence intervals), Figure S2 (bootstrap edge‐weight difference test). The case‐dropping bootstrap indicated good stability of strength centrality (CS coefficient = 0.52). Bootstrap‐based edge estimates and 95% confidence intervals are reported in Table S4. Stronger edges generally had narrower confidence intervals than weaker edges. For example, BIDQ_1–BIDQ_2 had a sample estimate of 0.494 with a 95% bootstrap confidence interval of 0.420–0.554, whereas BDDS_10–BDDS_11 had a sample estimate of 0.564 with a 95% bootstrap confidence interval of 0.436–0.679.

### 3.6 Network Comparison by Gender

**FIGURE 4:**
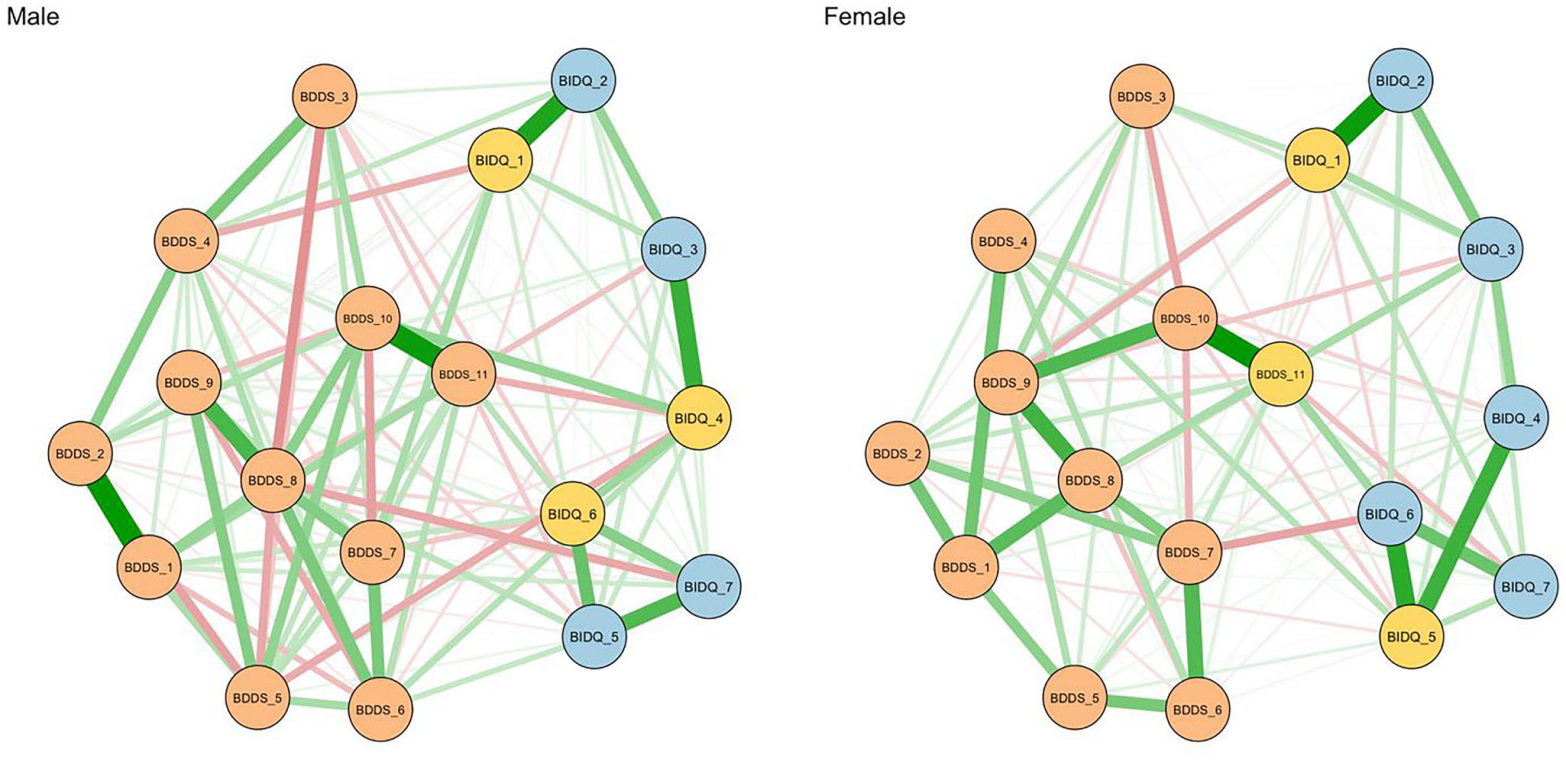
Gender‐specific BIDQ–BDDS‐5 symptom networks are displayed side‐by‐side using a common layout. Blue nodes indicate BIDQ items, orange nodes indicate BDDS‐5 items, and yellow nodes indicate the highest‐ranking bridge nodes within each gender‐specific network. Edge thickness corresponds to the magnitude of regularized partial correlations; green edges indicate positive associations and red edges indicate negative associations.

**FIGURE 5:**
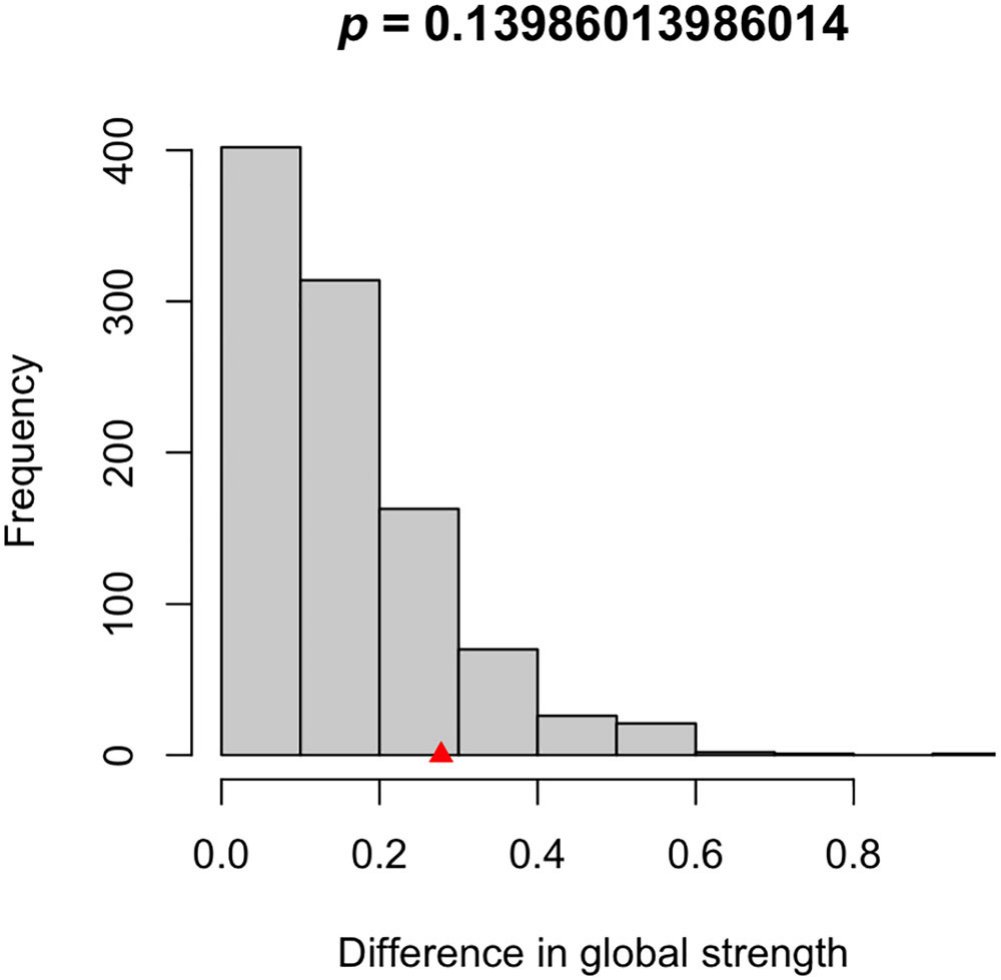
Permutation distribution of the difference in global strength between male and female BIDQ–BDDS‐5 symptom networks. The observed difference is indicated by the red triangle; the empirical *p*‐value was 0.140 based on 1000 permutations.

A comparison of the symptom networks stratified by gender showed both similarities and structural differences (Figure 4). Global strength was 7.86 for males and 8.13 for females, and this difference was not statistically significant (difference = 0.278, *p* = 0.140; Figure 5). In contrast, the test for network structure invariance was significant (*M* = 0.230, *p* = 0.041).

Gender‐specific bridge centrality profiles also differed. Among males, the highest bridge strength values were observed for BIDQ_4 (“Impairment in social/occupational functioning”; bridge strength = 0.885), BIDQ_6 (“Impact on academic/professional activities”; bridge strength = 0.738), and BIDQ_1 (“Concern about appearance of specific body part”; bridge strength = 0.634). Among females, the highest bridge strength values were observed for BIDQ_1 (“Concern about appearance of specific body part”; bridge strength = 0.579), BIDQ_5 (“Interference with social life”; bridge strength = 0.514), and BDDS_11 (“Trouble focusing due to appearance”; bridge strength = 0.508), followed by BIDQ_3 (“Emotional distress due to appearance concern”; bridge strength = 0.500) and BIDQ_6 (“Impact on academic/professional activities”; bridge strength = 0.496). Full bridge centrality estimates by gender are presented in Table S5.

A total of 19 edges showed statistically significant gender differences in edge weights (Table S6). Node strength invariance testing showed that only BDDS_5 (“Skin picking/clothing adjustment”) differed significantly by gender (*p* = 0.023), whereas the remaining nodes did not show significant strength differences (Table S7).

### 3.7 Sensitivity Analysis

A sensitivity analysis was conducted by re‐estimating the network with the additional inclusion of BDDS_12, the BDDS‐5 exclusion item related to weight‐ and eating‐disorder concerns. The overall pattern of findings remained largely stable. In the sensitivity model, BDDS_10, BDDS_7, BDDS_8, and BIDQ_6 remained among the most central nodes. BDDS_12 showed comparatively low strength (0.987), negative one‐step expected influence (−0.514), and low predictability (*R* ^2^ = 0.136; Table S8). Bridge‐centrality estimates in the sensitivity model were also similar to those in the primary model. BIDQ_6 continued to show the highest bridge strength (0.660), followed by BIDQ_1 (0.503), BIDQ_5 (0.352), BDDS_11 (0.318), and BIDQ_4 (0.315). BDDS_12 showed comparatively low bridge strength (0.103; Table S9).

Gender comparison results were similar across the primary and sensitivity models. In the primary network, the difference in global strength between males and females was not significant (difference = 0.278, *p* = 0.140), whereas network structure differed significantly (*M* = 0.230, *p* = 0.041). After adding BDDS_12, the global strength difference remained nonsignificant (difference = 0.294, *p* = 0.135), while the network structure difference remained significant (*M* = 0.233, *p* = 0.028; Table S10). The sensitivity analysis network is shown in Figure S4.

## 4 Discussion

The present study extends the literature by applying network analysis to examine the interplay between BID (BIDQ) and BDD (BDDS‐5) symptoms in a large sample of university students. By adopting a network perspective, our findings move beyond aggregate symptom scores and identify how specific symptoms are organized, interconnected, and differentially positioned within the broader symptom system. The estimated network indicated that BIDQ and BDDS‐5 symptoms formed distinct but interconnected communities, with several symptoms occupying influential central and bridge positions. These insights complement and extend a growing body of research using network models to examine the internal structure of psychopathology, particularly in relation to body image, eating‐related symptoms, and obsessive‐compulsive spectrum disorders. Importantly, the current findings provide evidence from a large Bangladeshi university sample, thereby expanding the cultural scope of the body image and dysmorphic symptom network literature, which has thus far been dominated by Western or clinical populations.

We found that BIDQ and BDDS‐5 items formed distinct but interconnected clusters, reflecting a modular organization consistent with earlier network studies. For example, Summers et al. (2020) showed that symptoms of BDD and major depressive disorder clustered within their respective domains but were linked by bridging symptoms, while Fuller‐Tyszkiewicz et al. (2020) identified separate clusters of body dissatisfaction features, with the centrality of specific concerns, such as weight and shape, varying by group. A network analysis of transgender individuals with gender dysphoria also found evidence of modularity, where satisfaction with body regions such as chest, hair, voice, and muscularity formed interconnected clusters (Van de Grift et al. 2016). Genital dissatisfaction, however, was highly isolated and the most peripheral node in that network, highlighting its distinct psychological role. In contrast, socially salient features, such as hair, voice, posture, and muscularity, were more central, suggesting that these attributes may have greater influence on overall body satisfaction and distress than more isolated body‐related concerns (Van de Grift et al. 2016). Our network reflected a similar pattern of modularity, with BIDQ and BDDS‐5 symptoms clustering primarily within their respective domains and cross‐domain connections generally being weaker than the strongest within‐community edges. This pattern supports the interpretation that BID and body dysmorphic symptoms are related but not interchangeable dimensions of body‐image‐related psychopathology. In addition, we observed both positive and negative associations among symptoms. However, the negative edges were generally smaller in magnitude and should be interpreted cautiously because they reflect conditional relations estimated after adjustment for all other symptoms in the network rather than simple protective effects. In this context, such edges may indicate inverse conditional relations or suppression effects rather than straightforward symptom buffering.

Our network identified social comparison, academic/professional impact, and appearance‐related emotional distress as the most central symptoms in the final network, with feeling ugly/strange and difficulty doing things with others also showing high centrality. In contrast, Cerea et al. (2024) found that body appreciation and functionality appreciation—dimensions of positive body image—were the most central nodes in a network integrating eating disorder symptoms and protective factors, emphasizing the protective role of positive body image in reducing eating pathology. Among eating disorder symptoms, drive for thinness and body dissatisfaction were the most influential. Similarly, a study identified interference in functioning, worthlessness, and loss of pleasure as core symptoms in the joint network of body dysmorphic and depressive symptoms (Summers et al. 2020), while Finch et al. (2023) observed that body dissatisfaction and daily functioning impairment served as central and bridging features in networks of eating pathology and premenstrual symptoms. Thus, although the specific content of the most central symptoms differs across studies, a recurring theme is that functional impairment and affectively salient symptoms often occupy influential positions within symptom networks. In the present study, bridge centrality analysis further showed that academic/professional impact, concern about a specific appearance feature, and social interference were the strongest connectors between BIDQ and BDDS‐5 domains. Among the BDDS‐5 items, trouble focusing due to appearance, feeling bad or miserable about appearance, and perceiving that others do not think anything is wrong showed the highest bridge strength values. These findings suggest that, within body image and dysmorphic symptom networks, the most central nodes and key bridge symptoms may vary across contexts. In particular, the prominence of academic/professional impact suggests that appearance‐related distress may become especially relevant when it interferes with academic role functioning. Similarly, the bridge roles of specific appearance concern and social interference suggest that focused appearance worries and interpersonal consequences may be important pathways linking broader body image dissatisfaction with dysmorphic symptom processes.

Studies from diverse contexts consistently highlight gender differences in body image and dysmorphic symptoms. For example, Taqui et al. (2008) found that BDD was more prevalent among male than female medical students in Pakistan, with males most concerned about hair and thinness and females focusing more on fatness, skin, and teeth. In contrast, a large multicenter dermatological study demonstrated that dysmorphic concern and BDD were more common in women with skin conditions across Europe, with women reporting higher distress levels and greater vulnerability to stigma, particularly in conditions such as vitiligo (Sampogna et al. 2024). These findings suggest that gender differences in the prevalence and focus of BDD symptoms may occur across both clinical and nonclinical populations and are shaped by sociocultural context. Consistent with these broader patterns, our gender‐stratified network analysis revealed both shared and unique features. While overall connectivity, as indicated by global strength, did not differ significantly between males and females, the network structure differed significantly, indicating that the configuration of symptom interrelations varied by gender. For males, the strongest bridge symptoms were impairment in social/occupational functioning, academic/professional impact, and concern about a specific appearance feature. In contrast, among females, the strongest bridge symptoms included concern about a specific appearance feature, social interference, trouble focusing due to appearance, and emotional distress related to appearance concerns. Moreover, 19 edges showed nominally significant gender differences in association strength, further suggesting the presence of gender‐specific pathways of symptom co‐occurrence. Centrality invariance analysis further indicated that most nodes did not differ significantly in strength across genders, although skin picking/clothing adjustment showed a significant gender difference in node strength.

These gender‐specific patterns may be understood in light of broader sociocultural expectations and role demands. In the context of South Asian gender norms, the prominence of functional impairment‐related bridge symptoms in males may reflect the greater salience of competence, performance, and role functioning, whereas the stronger bridging role of specific appearance concern, social interference, concentration‐related burden, and emotional distress in females may reflect heightened sensitivity to appearance evaluation and interpersonal presentation. Although this interpretation remains tentative, it is consistent with prior evidence showing that body image experiences are shaped not only by gender but also by the social meanings attached to appearance and functioning within a given cultural context. Although few studies have conducted gender‐stratified network analyses of body image or BDD symptoms, existing evidence and our findings suggest that meaningful differences in network structure and centrality may exist across different groups. For example, Naraindas et al. (2025) used network analysis to show that the centrality and connectivity of self‐objectification and interoceptive awareness features varied according to body dissatisfaction levels among women, illustrating how network approaches can reveal subgroup‐specific psychological targets. Fuller‐Tyszkiewicz et al. (2020) further showed that symptom centrality can shift with population characteristics, such as pregnancy status, reinforcing the need for more detailed subgroup analyses in future research. Thus, these findings emphasize the importance of gender‐sensitive assessment and intervention strategies that account for both shared and unique symptom dynamics across populations.

The findings of our study may have implications for prevention and intervention efforts targeting BID and body dysmorphic symptoms among university students. By identifying social comparison, academic/professional impact, and appearance‐related emotional distress as highly central symptoms, and academic/professional impact, specific appearance concern, social interference, and trouble focusing due to appearance as important bridge symptoms, our results may help inform targeted prevention and intervention priorities. In particular, the prominence of academic/professional impact suggests that body‐image‐related distress in university settings may be especially consequential when it interferes with concentration, study engagement, confidence, and everyday role functioning. Likewise, the strong bridge roles of specific appearance concern and social interference suggest that interventions may benefit from addressing both focused appearance worry and the interpersonal consequences of that distress. The prominence of social and functional impairment also underscores the potential value of campus‐based programs, cognitive‐behavioral strategies, and supportive environments that address not only appearance concerns but also everyday functioning and well‐being. More specifically, university‐based interventions may benefit from integrating body image support with academic counseling, stress management, and psychosocial services, particularly for students whose appearance concerns are accompanied by avoidance, impaired concentration, or reduced academic performance.

Importantly, our gender‐stratified analysis revealed that while some symptoms were influential across both males and females, the configuration of bridge symptoms and specific pathways of symptom co‐occurrence differed by gender. This highlights the potential value of gender‐sensitive and tailored approaches that consider distinct symptom dynamics in male and female students. For male students, assessment and intervention efforts may need to pay particular attention to symptoms related to role impairment and academic/professional functioning. For female students, stronger emphasis may be placed on specific appearance concern, social interference, emotional distress, and concentration‐related burden linked to appearance concerns. Recent advances in network intervention research, such as Bernstein et al. (2023), suggest that CBT for BDD may influence central symptoms, with changes in these key nodes associated with broader improvements across symptom networks. Although our cross‐sectional design precludes causal interpretation, these findings support the value of considering central and bridge symptoms—including those particularly salient for each gender—as potential targets for future intervention research. Overall, this underscores the utility of a network approach for identifying influential symptoms and informing targeted, symptom‐level, and context‐sensitive prevention and intervention strategies for body image and dysmorphic problems in university settings, with attention to gender‐specific needs.

A major strength of this study is its application of network analysis to examine the interplay between BID and BDD symptoms in a large sample of university students. Item‐level analysis enabled the identification of both central and bridge symptoms, offering nuanced insights beyond traditional sum‐score approaches. Stratifying the networks by gender further allowed exploration of shared and unique symptom dynamics across males and females, providing an empirical basis for gender‐sensitive interpretation. An additional strength is that the main findings were supported by a sensitivity analysis using an alternative network specification, indicating that the overall interpretation was robust to inclusion of the BDDS‐5 exclusion item related to weight‐ and eating‐disorder concerns. However, several limitations should be acknowledged. The cross‐sectional design precludes inference regarding causality or temporal relationships, limiting conclusions about symptom directionality. Accordingly, the network should be interpreted as representing conditional associations among symptoms rather than causal pathways or temporal processes. Reliance on self‐report questionnaires may introduce biases such as social desirability or recall error. In addition, because the data were derived from screening instruments rather than clinical interviews, the findings should not be interpreted as equivalent to clinical diagnosis. Although our sample was relatively large, it was limited to university students recruited from dormitories in a single Bangladeshi university setting, which may restrict generalizability. Moreover, participants were recruited through convenience sampling from university dormitories, which may have overrepresented students exposed to relatively dense peer interaction and shared campus‐related social experiences. This sampling context may be particularly relevant when interpreting the prominence of symptoms related to social interference and academic/professional functioning. While network analysis is useful for identifying key symptoms, it does not directly account for potential confounders or the underlying latent structure of the disorders. Finally, although we conducted gender‐stratified analyses, other important demographic or psychosocial factors—such as social media exposure, depressive symptoms, anxiety, eating‐related psychopathology, or other psychosocial stressors—were not examined and warrant investigation in future research.

## 5 Conclusions

This study showed that BID and body dysmorphic symptoms formed distinct but interconnected symptom communities among Bangladeshi university students. Social comparison, academic/professional impact, and appearance‐related emotional distress emerged as highly central symptoms, while academic/professional impact, concern about a specific appearance feature, and social interference were the most prominent bridge symptoms linking the BIDQ and BDDS‐5 domains. Although overall network connectivity did not differ significantly by gender, the structure of symptom interrelations and bridge profiles varied between male and female students, suggesting potentially gender‐specific patterns of symptom co‐occurrence. These findings extend symptom‐level body image and dysmorphic symptom research to a large non‐Western university sample and highlight the value of moving beyond aggregate scale scores. Overall, the findings may inform targeted and gender‐sensitive prevention or intervention strategies in university settings, particularly where appearance‐related distress affects social functioning, concentration, and academic role performance. Given the cross‐sectional design, these findings should be interpreted as conditional associations rather than causal pathways.

## Author Contributions

**Mohammed A. Mamun**: conceptualization, investigation, writing – original draft, writing – review and editing, visualization, validation, methodology, software, formal analysis, project administration, resources. **S. M. Rakibul Hasan**: investigation, methodology, data curation, project administration. **Moneerah Mohammad ALmerab**: supervision, validation, visualization, writing – review and editing, methodology. **Firoj Al‐Mamun**: conceptualization, investigation, methodology, validation, visualization, writing – review and editing, software, data curation, project administration.

## Funding

Dr ALmerab is currently receiving funding support from the Princess Nourah Bint Abdulrahman University Researchers Supporting Project (Number PNURSP2026R563), Princess Nourah Bint Abdulrahman University, Riyadh, Saudi Arabia.

## Ethics Statement

The authors affirm that all procedures contributing to this research adhered to the ethical standards of the relevant national and institutional committees governing human research, as well as the principles outlined in the Declaration of Helsinki (1975, revised 2013). Ethical approval for all study procedures involving human participants was obtained from the ethics committee at CHINTA Research Bangladesh (Reference: CHINTA/IRB/10‐2024/17).

## Consent

Before initiating the survey, participants were fully informed about the study's objectives, procedures, potential risks, and their rights—including the right to withdraw from the study at any time without penalty. Written informed consent was obtained from all participants to confirm their understanding and voluntary participation. To protect confidentiality, all data were securely stored and accessible only to the research team and were used solely for research purposes. No monetary or non‐monetary incentives were provided for participation.

## Conflicts of Interest

The authors declare no conflicts of interest.

## Supporting information




**Supplementary Materials**: brb371538‐sup‐0001‐SuppMat.docx

## Data Availability

The data that support the findings of this study are available from the corresponding author upon reasonable request.
