# Supplementary material for: Bridging Body Image Disturbance and Body Dysmorphic Disorder Symptoms: A Symptom Network Study Among University Students
Source: Brain Behav. 2026 Jun 8;16(6):e71538. doi: 10.1002/brb3.71538 (PMC13247133; doi:10.1002/brb3.71538)

# Supplementary Materials

# Bridging body image disturbance and body dysmorphic disorder symptoms: a symptom network study among university student

Mamun et al. (2026), Brain and Behavior, email: [mamun@thechinta.org](mailto:mamun@thechinta.org)

# Supplementary Tables and Figures

This document presents supplementary tables and figures for the symptom network analysis of body image disturbance and body dysmorphic symptoms.

## Supplementary Table S0. Scale Content and Symptom Mapping

Item-level mapping of the Body Image Disturbance Questionnaire (BIDQ) and the Body Dysmorphic Disorder Screener for DSM-5 (BDDS-5), including scale membership and symptom labels used in the network analysis.

| Item | Scale | Construct_Label |
| --- | --- | --- |
| BIDQ_1 | BIDQ | Concern about appearance of specific body part |
| BIDQ_2 | BIDQ | Preoccupation with perceived defect |
| BIDQ_3 | BIDQ | Emotional distress due to appearance concern |
| BIDQ_4 | BIDQ | Impairment in social/occupational functioning |
| BIDQ_5 | BIDQ | Interference with social life |
| BIDQ_6 | BIDQ | Impact on academic/professional activities |
| BIDQ_7 | BIDQ | Avoidance behaviors |
| BDDS_1 | BDDS-5 | I look strange or ugly |
| BDDS_2 | BDDS-5 | I constantly think about appearance |
| BDDS_3 | BDDS-5 | Others do not think anything is wrong |
| BDDS_4 | BDDS-5 | Mirror checking / mirror avoidance |
| BDDS_5 | BDDS-5 | Skin picking / clothing adjustment |
| BDDS_6 | BDDS-5 | Reassurance seeking |
| BDDS_7 | BDDS-5 | Comparing appearance with others |
| BDDS_8 | BDDS-5 | Feel bad or miserable about appearance |
| BDDS_9 | BDDS-5 | Avoid activities due to appearance |
| BDDS_10 | BDDS-5 | Difficult doing things with others |
| BDDS_11 | BDDS-5 | Trouble focusing due to appearance |

## Supplementary Table S1. Edge Weights

Regularized partial correlation matrix showing edge weights among BIDQ and BDDS-5 symptom nodes in the estimated network.

| Node | BIDQ_1 | BIDQ_2 | BIDQ_3 | BIDQ_4 | BIDQ_5 | BIDQ_6 | BIDQ_7 | BDDS_1 | BDDS_2 | BDDS_3 | BDDS_4 | BDDS_5 | BDDS_6 | BDDS_7 | BDDS_8 | BDDS_9 | BDDS_10 | BDDS_11 |
| --- | --- | --- | --- | --- | --- | --- | --- | --- | --- | --- | --- | --- | --- | --- | --- | --- | --- | --- |
| BIDQ_1 | 0.00000000 | 0.493572487 | 0.1904248457 | 0.020422329 | 0.039217770 | -0.03686057 | 0.095956121 | -0.01057557 | 0.06313937 | 0.0241611315 | -0.104046916 | 0.0724345933 | 0.066178029 | 0.09327303 | 0.01119908 | -0.078611168 | 0.0000000000 | -0.053798758 |
| BIDQ_2 | 0.49357249 | 0.000000000 | 0.2421371009 | 0.060239600 | 0.000000000 | 0.09479968 | 0.052167137 | 0.00000000 | 0.04220627 | 0.0223357162 | 0.083357865 | 0.0069891035 | -0.009823472 | 0.03386796 | -0.01752167 | -0.011505295 | 0.0000000000 | -0.036941330 |
| BIDQ_3 | 0.19042485 | 0.242137101 | 0.0000000000 | 0.335218899 | 0.097445478 | -0.02833183 | 0.117344736 | -0.03238526 | 0.00000000 | 0.0999069066 | 0.002393402 | -0.0259841875 | 0.000000000 | -0.01026065 | 0.09435429 | -0.019199877 | -0.0001182197 | 0.000000000 |
| BIDQ_4 | 0.02042233 | 0.060239600 | 0.3352188993 | 0.000000000 | 0.272110902 | 0.17136247 | 0.000000000 | 0.00000000 | 0.01734425 | 0.0000000000 | 0.000000000 | -0.0719671811 | 0.078736477 | 0.00000000 | 0.02033474 | 0.007517532 | 0.1088141186 | -0.099705329 |
| BIDQ_5 | 0.03921777 | 0.000000000 | 0.0974454775 | 0.272110902 | 0.000000000 | 0.34676812 | 0.280847657 | -0.07697656 | 0.00000000 | -0.1044774370 | 0.000000000 | 0.0864961833 | 0.026422055 | 0.00000000 | 0.08955152 | 0.000000000 | -0.0530059697 | -0.005402298 |
| BIDQ_6 | -0.03686057 | 0.094799678 | -0.0283318305 | 0.171362472 | 0.346768117 | 0.00000000 | 0.275847074 | 0.04296917 | 0.00000000 | -0.0591889622 | 0.047451100 | 0.0470567303 | -0.067028327 | -0.14725708 | -0.07311770 | 0.099086407 | 0.0000000000 | 0.173414688 |
| BIDQ_7 | 0.09595612 | 0.052167137 | 0.1173447361 | 0.000000000 | 0.280847657 | 0.27584707 | 0.000000000 | 0.08462530 | 0.00000000 | 0.0430200247 | 0.000000000 | 0.0000000000 | 0.000000000 | 0.01747537 | -0.05116434 | 0.004108775 | 0.0207708842 | -0.020462994 |
| BDDS_1 | -0.01057557 | 0.000000000 | -0.0323852610 | 0.000000000 | -0.076976557 | 0.04296917 | 0.084625302 | 0.00000000 | 0.42985921 | 0.0000000000 | 0.224820795 | 0.2300992663 | -0.081651915 | 0.01936543 | 0.26733719 | 0.000000000 | 0.0000000000 | 0.068148888 |
| BDDS_2 | 0.06313937 | 0.042206273 | 0.0000000000 | 0.017344253 | 0.000000000 | 0.00000000 | 0.000000000 | 0.42985921 | 0.00000000 | 0.0000000000 | 0.178929997 | -0.1050861160 | -0.074392363 | 0.16021326 | 0.00000000 | 0.071587014 | 0.1423383997 | 0.017634263 |
| BDDS_3 | 0.02416113 | 0.022335716 | 0.0999069066 | 0.000000000 | -0.104477437 | -0.05918896 | 0.043020025 | 0.00000000 | 0.00000000 | 0.0000000000 | 0.228796224 | 0.0003764997 | 0.045929439 | 0.06297789 | -0.08273624 | 0.045632014 | 0.0000000000 | 0.000000000 |
| BDDS_4 | -0.10404692 | 0.083357865 | 0.0023934021 | 0.000000000 | 0.000000000 | 0.04745110 | 0.000000000 | 0.22482080 | 0.17893000 | 0.2287962236 | 0.000000000 | 0.0152528820 | 0.144020990 | -0.06284819 | 0.08478752 | 0.040946709 | 0.1136192214 | 0.015062676 |
| BDDS_5 | 0.07243459 | 0.006989103 | -0.0259841875 | -0.071967181 | 0.086496183 | 0.04705673 | 0.000000000 | 0.23009927 | -0.10508612 | 0.0003764997 | 0.015252882 | 0.0000000000 | 0.231810765 | 0.14633573 | -0.05314266 | 0.172384861 | 0.0733063870 | 0.123644166 |
| BDDS_6 | 0.06617803 | -0.009823472 | 0.0000000000 | 0.078736477 | 0.026422055 | -0.06702833 | 0.000000000 | -0.08165191 | -0.07439236 | 0.0459294389 | 0.144020990 | 0.2318107648 | 0.000000000 | 0.30701524 | 0.09826028 | -0.071047746 | 0.0345612986 | 0.048103336 |
| BDDS_7 | 0.09327303 | 0.033867963 | -0.0102606512 | 0.000000000 | 0.000000000 | -0.14725708 | 0.017475372 | 0.01936543 | 0.16021326 | 0.0629778861 | -0.062848186 | 0.1463357328 | 0.307015238 | 0.00000000 | 0.23912443 | 0.131111837 | -0.2160431704 | 0.175166022 |
| BDDS_8 | 0.01119908 | -0.017521673 | 0.0943542913 | 0.020334739 | 0.089551524 | -0.07311770 | -0.051164344 | 0.26733719 | 0.00000000 | -0.0827362380 | 0.084787517 | -0.0531426551 | 0.098260278 | 0.23912443 | 0.00000000 | 0.344932471 | 0.1211622606 | 0.000000000 |
| BDDS_9 | -0.07861117 | -0.011505295 | -0.0191998774 | 0.007517532 | 0.000000000 | 0.09908641 | 0.004108775 | 0.00000000 | 0.07158701 | 0.0456320136 | 0.040946709 | 0.1723848607 | -0.071047746 | 0.13111184 | 0.34493247 | 0.000000000 | 0.1183016408 | 0.000000000 |
| BDDS_10 | 0.00000000 | 0.000000000 | -0.0001182197 | 0.108814119 | -0.053005970 | 0.00000000 | 0.020770884 | 0.00000000 | 0.14233840 | 0.0000000000 | 0.113619221 | 0.0733063870 | 0.034561299 | -0.21604317 | 0.12116226 | 0.118301641 | 0.0000000000 | 0.563619256 |
| BDDS_11 | -0.05379876 | -0.036941330 | 0.0000000000 | -0.099705329 | -0.005402298 | 0.17341469 | -0.020462994 | 0.06814889 | 0.01763426 | 0.0000000000 | 0.015062676 | 0.1236441656 | 0.048103336 | 0.17516602 | 0.00000000 | 0.000000000 | 0.5636192559 | 0.000000000 |

## Supplementary Table S2. Centrality Indices

Node-wise centrality estimates for the estimated network, including strength, one-step expected influence, two-step expected influence, and predictability.

| Node | Strength | ExpectedInfluence_1step | ExpectedInfluence_2step | Predictability_R2 | ItemText |
| --- | --- | --- | --- | --- | --- |
| BIDQ_1 | 1.4538718 | 0.8860858 | 1.7747815 | 0.33543035 | Concern about appearance of specific body part |
| BIDQ_2 | 1.2074647 | 1.0558812 | 2.0360841 | 0.32990659 | Preoccupation with perceived defect |
| BIDQ_3 | 1.2955057 | 1.0629456 | 2.0256402 | 0.25241957 | Emotional distress due to appearance concern |
| BIDQ_4 | 1.2637738 | 0.9204288 | 1.8353543 | 0.25375824 | Impairment in social/occupational functioning |
| BIDQ_5 | 1.4787219 | 0.9989974 | 1.9709565 | 0.32008046 | Interference with social life |
| BIDQ_6 | 1.7105399 | 0.8869710 | 1.7867016 | 0.31808325 | Impact on academic/professional activities |
| BIDQ_7 | 1.0637904 | 0.9205357 | 1.7889938 | 0.19346818 | Avoidance behaviors |
| BDDS_1 | 1.5688146 | 1.1656359 | 2.3261153 | 0.38751833 | I look strange or ugly |
| BDDS_2 | 1.3027305 | 0.9437736 | 1.9614970 | 0.29080515 | I constantly think about appearance |
| BDDS_3 | 0.8195385 | 0.3267332 | 0.6367314 | 0.09468477 | Others do not think anything is wrong |
| BDDS_4 | 1.3463345 | 1.0125443 | 1.8844491 | 0.20186603 | Mirror checking / mirror avoidance |
| BDDS_5 | 1.4623673 | 0.9500070 | 1.8439297 | 0.21342032 | Skin picking / clothing adjustment |
| BDDS_6 | 1.3849817 | 0.7770941 | 1.5007259 | 0.21712507 | Reassurance seeking |
| BDDS_7 | 1.8223353 | 0.9495171 | 1.7720917 | 0.33330273 | Comparing appearance with others |
| BDDS_8 | 1.6487264 | 1.0933612 | 2.2241393 | 0.31455393 | Feel bad or miserable about appearance |
| BDDS_9 | 1.2159733 | 0.8552452 | 1.7072716 | 0.21038417 | Avoid activities due to appearance |
| BDDS_10 | 1.5656608 | 1.0273261 | 2.0137721 | 0.44783633 | Difficult doing things with others |
| BDDS_11 | 1.4011040 | 0.9684826 | 1.9311337 | 0.41585443 | Trouble focusing due to appearance |

## Supplementary Table S3. Bridge Centrality

Bridge centrality estimates for BIDQ and BDDS-5 symptom nodes, including bridge strength, one-step bridge expected influence, and two-step bridge expected influence.

| Node | Community | BridgeStrength | BridgeEI_1step | BridgeEI_2step | ItemText |
| --- | --- | --- | --- | --- | --- |
| BIDQ_1 | BIDQ | 0.5774177 | 0.083352823 | 0.19655337 | Concern about appearance of specific body part |
| BIDQ_2 | BIDQ | 0.2645487 | 0.112965151 | 0.28527076 | Preoccupation with perceived defect |
| BIDQ_3 | BIDQ | 0.2846028 | 0.108706403 | 0.22156890 | Emotional distress due to appearance concern |
| BIDQ_4 | BIDQ | 0.4044196 | 0.061074610 | 0.14438783 | Impairment in social/occupational functioning |
| BIDQ_5 | BIDQ | 0.4423320 | -0.037392499 | 0.04782543 | Interference with social life |
| BIDQ_6 | BIDQ | 0.7565702 | 0.063386023 | 0.20843107 | Impact on academic/professional activities |
| BIDQ_7 | BIDQ | 0.2416277 | 0.098373019 | 0.21015377 | Avoidance behaviors |
| BDDS_1 | BDDS | 0.2475319 | 0.007657083 | 0.08801020 | I look strange or ugly |
| BDDS_2 | BDDS | 0.1226899 | 0.122689900 | 0.22562740 | I constantly think about appearance |
| BDDS_3 | BDDS | 0.3530902 | 0.025757380 | 0.04410353 | Others do not think anything is wrong |
| BDDS_4 | BDDS | 0.2372493 | 0.029155452 | 0.12567944 | Mirror checking / mirror avoidance |
| BDDS_5 | BDDS | 0.3109280 | 0.115025242 | 0.22748624 | Skin picking / clothing adjustment |
| BDDS_6 | BDDS | 0.2481884 | 0.094484762 | 0.20423610 | Reassurance seeking |
| BDDS_7 | BDDS | 0.3021341 | -0.012901371 | 0.03639802 | Comparing appearance with others |
| BDDS_8 | BDDS | 0.3572434 | 0.073635916 | 0.17636199 | Feel bad or miserable about appearance |
| BDDS_9 | BDDS | 0.2200291 | 0.001396373 | 0.05758837 | Avoid activities due to appearance |
| BDDS_10 | BDDS | 0.1827092 | 0.076460813 | 0.15217109 | Difficult doing things with others |
| BDDS_11 | BDDS | 0.3897254 | -0.042896021 | -0.02347123 | Trouble focusing due to appearance |

## Supplementary Table S4. Bootstrap Confidence Intervals

Bootstrap-based estimates for network edge weights, including sample estimates, bootstrap means, and 95% confidence intervals.

| Edge | SampleEstimate | BootstrapMean | CI_lower | CI_upper |
| --- | --- | --- | --- | --- |
| BIDQ_1 -- BIDQ_2 | 0.4935724871 | 0.4886542469 | 0.420038847 | 0.554363241 |
| BIDQ_1 -- BIDQ_3 | 0.1904248457 | 0.1870044575 | 0.103481808 | 0.266334046 |
| BIDQ_2 -- BIDQ_3 | 0.2421371009 | 0.2437377476 | 0.168152320 | 0.315941940 |
| BIDQ_1 -- BIDQ_4 | 0.0204223290 | 0.0243226332 | -0.024447382 | 0.098351531 |
| BIDQ_2 -- BIDQ_4 | 0.0602396000 | 0.0574602035 | 0.000000000 | 0.134238179 |
| BIDQ_3 -- BIDQ_4 | 0.3352188993 | 0.3296917600 | 0.243467372 | 0.408801580 |
| BIDQ_1 -- BIDQ_5 | 0.0392177701 | 0.0409964470 | 0.000000000 | 0.123921306 |
| BIDQ_2 -- BIDQ_5 | 0.0000000000 | 0.0024197776 | -0.068165851 | 0.075100019 |
| BIDQ_3 -- BIDQ_5 | 0.0974454775 | 0.1007377603 | 0.018246565 | 0.182004528 |
| BIDQ_4 -- BIDQ_5 | 0.2721109025 | 0.2667355404 | 0.173461605 | 0.353513417 |
| BIDQ_1 -- BIDQ_6 | -0.0368605723 | -0.0358725397 | -0.125576987 | 0.014297059 |
| BIDQ_2 -- BIDQ_6 | 0.0947996778 | 0.1006054674 | 0.011467390 | 0.194188080 |
| BIDQ_3 -- BIDQ_6 | -0.0283318305 | -0.0341489197 | -0.128720531 | 0.016437144 |
| BIDQ_4 -- BIDQ_6 | 0.1713624725 | 0.1759135940 | 0.076732160 | 0.275112135 |
| BIDQ_5 -- BIDQ_6 | 0.3467681175 | 0.3499649893 | 0.251287733 | 0.447624562 |
| BIDQ_1 -- BIDQ_7 | 0.0959561206 | 0.0937502947 | 0.014589445 | 0.169797694 |
| BIDQ_2 -- BIDQ_7 | 0.0521671368 | 0.0506374465 | 0.000000000 | 0.130322556 |
| BIDQ_3 -- BIDQ_7 | 0.1173447361 | 0.1169458349 | 0.039925005 | 0.194111081 |
| BIDQ_4 -- BIDQ_7 | 0.0000000000 | 0.0077243025 | -0.049570136 | 0.074784162 |
| BIDQ_5 -- BIDQ_7 | 0.2808476566 | 0.2752901728 | 0.173704277 | 0.372595700 |
| BIDQ_6 -- BIDQ_7 | 0.2758470744 | 0.2752443692 | 0.182586702 | 0.368015405 |
| BIDQ_1 -- BDDS_1 | -0.0105755745 | -0.0241732683 | -0.131320632 | 0.056657938 |
| BIDQ_2 -- BDDS_1 | 0.0000000000 | -0.0021062865 | -0.092422446 | 0.082806542 |
| BIDQ_3 -- BDDS_1 | -0.0323852610 | -0.0463533909 | -0.166827002 | 0.033033415 |
| BIDQ_4 -- BDDS_1 | 0.0000000000 | 0.0113333266 | -0.086490977 | 0.119319101 |
| BIDQ_5 -- BDDS_1 | -0.0769765569 | -0.0761338158 | -0.205265894 | 0.000000000 |
| BIDQ_6 -- BDDS_1 | 0.0429691738 | 0.0532913568 | -0.034300010 | 0.177413185 |
| BIDQ_7 -- BDDS_1 | 0.0846253020 | 0.0873728713 | 0.000000000 | 0.204472340 |
| BIDQ_1 -- BDDS_2 | 0.0631393741 | 0.0725703715 | -0.004152322 | 0.199807254 |
| BIDQ_2 -- BDDS_2 | 0.0422062730 | 0.0514238844 | -0.017165299 | 0.153729588 |
| BIDQ_3 -- BDDS_2 | 0.0000000000 | 0.0098764321 | -0.086974925 | 0.118930063 |
| BIDQ_4 -- BDDS_2 | 0.0173442529 | 0.0246207054 | -0.061479305 | 0.126167284 |
| BIDQ_5 -- BDDS_2 | 0.0000000000 | -0.0101305081 | -0.119365050 | 0.102299400 |
| BIDQ_6 -- BDDS_2 | 0.0000000000 | -0.0153235600 | -0.134502534 | 0.078651175 |
| BIDQ_7 -- BDDS_2 | 0.0000000000 | -0.0046475698 | -0.113233313 | 0.096587297 |
| BDDS_1 -- BDDS_2 | 0.4298592070 | 0.4364281548 | 0.305151649 | 0.562836744 |
| BIDQ_1 -- BDDS_3 | 0.0241611315 | 0.0281037261 | -0.040946692 | 0.115378428 |
| BIDQ_2 -- BDDS_3 | 0.0223357162 | 0.0253082825 | -0.039180601 | 0.108288573 |
| BIDQ_3 -- BDDS_3 | 0.0999069066 | 0.0929325521 | 0.000000000 | 0.187222197 |
| BIDQ_4 -- BDDS_3 | 0.0000000000 | 0.0104591243 | -0.070965087 | 0.099749488 |
| BIDQ_5 -- BDDS_3 | -0.1044774370 | -0.1057628821 | -0.204650026 | -0.004503802 |
| BIDQ_6 -- BDDS_3 | -0.0591889622 | -0.0659428801 | -0.172101154 | 0.011809715 |
| BIDQ_7 -- BDDS_3 | 0.0430200247 | 0.0443389927 | -0.020199023 | 0.132886435 |
| BDDS_1 -- BDDS_3 | 0.0000000000 | 0.0057138036 | -0.121919812 | 0.127774845 |
| BDDS_2 -- BDDS_3 | 0.0000000000 | -0.0098869871 | -0.136174831 | 0.112198334 |
| BIDQ_1 -- BDDS_4 | -0.1040469158 | -0.1061555357 | -0.234507851 | 0.000000000 |
| BIDQ_2 -- BDDS_4 | 0.0833578655 | 0.0847279565 | 0.000000000 | 0.205395743 |
| BIDQ_3 -- BDDS_4 | 0.0023934021 | 0.0227545822 | -0.065889445 | 0.134561487 |
| BIDQ_4 -- BDDS_4 | 0.0000000000 | -0.0238382935 | -0.125443672 | 0.057868464 |
| BIDQ_5 -- BDDS_4 | 0.0000000000 | 0.0086886248 | -0.083275475 | 0.113574969 |
| BIDQ_6 -- BDDS_4 | 0.0474511004 | 0.0552041179 | -0.034932431 | 0.168959667 |
| BIDQ_7 -- BDDS_4 | 0.0000000000 | -0.0199801896 | -0.139919158 | 0.068930685 |
| BDDS_1 -- BDDS_4 | 0.2248207953 | 0.2217865139 | 0.065219083 | 0.369640538 |
| BDDS_2 -- BDDS_4 | 0.1789299969 | 0.1804600007 | 0.017820404 | 0.331756310 |
| BDDS_3 -- BDDS_4 | 0.2287962236 | 0.2275756098 | 0.104834417 | 0.364709624 |
| BIDQ_1 -- BDDS_5 | 0.0724345933 | 0.0728139843 | 0.000000000 | 0.174877437 |
| BIDQ_2 -- BDDS_5 | 0.0069891035 | 0.0231014769 | -0.040526789 | 0.106206409 |
| BIDQ_3 -- BDDS_5 | -0.0259841875 | -0.0364629658 | -0.139828059 | 0.033085016 |
| BIDQ_4 -- BDDS_5 | -0.0719671811 | -0.0833981968 | -0.206434786 | 0.000000000 |
| BIDQ_5 -- BDDS_5 | 0.0864961833 | 0.0881044477 | 0.000000000 | 0.204048173 |
| BIDQ_6 -- BDDS_5 | 0.0470567303 | 0.0487215483 | -0.041490141 | 0.170174762 |
| BIDQ_7 -- BDDS_5 | 0.0000000000 | 0.0082173767 | -0.083452577 | 0.107145389 |
| BDDS_1 -- BDDS_5 | 0.2300992663 | 0.2280033343 | 0.064041985 | 0.382840164 |
| BDDS_2 -- BDDS_5 | -0.1050861160 | -0.1139040892 | -0.296330757 | 0.009543970 |
| BDDS_3 -- BDDS_5 | 0.0003764997 | 0.0100743688 | -0.105657551 | 0.123133891 |
| BDDS_4 -- BDDS_5 | 0.0152528820 | 0.0268847414 | -0.111236192 | 0.181637156 |
| BIDQ_1 -- BDDS_6 | 0.0661780286 | 0.0678404932 | 0.000000000 | 0.165643390 |
| BIDQ_2 -- BDDS_6 | -0.0098234716 | -0.0252566844 | -0.109273892 | 0.035306900 |
| BIDQ_3 -- BDDS_6 | 0.0000000000 | 0.0103695260 | -0.075379822 | 0.105137853 |
| BIDQ_4 -- BDDS_6 | 0.0787364775 | 0.0766854783 | 0.000000000 | 0.181357523 |
| BIDQ_5 -- BDDS_6 | 0.0264220554 | 0.0382620380 | -0.038144116 | 0.138170329 |
| BIDQ_6 -- BDDS_6 | -0.0670283275 | -0.0754744911 | -0.186812308 | 0.000000000 |
| BIDQ_7 -- BDDS_6 | 0.0000000000 | -0.0003434526 | -0.086118675 | 0.085306157 |
| BDDS_1 -- BDDS_6 | -0.0816519149 | -0.0902109562 | -0.256626454 | 0.022092322 |
| BDDS_2 -- BDDS_6 | -0.0743923634 | -0.0771626556 | -0.240849515 | 0.052023647 |
| BDDS_3 -- BDDS_6 | 0.0459294389 | 0.0485905453 | -0.055170373 | 0.170118613 |
| BDDS_4 -- BDDS_6 | 0.1440209904 | 0.1482664551 | 0.000000000 | 0.299165948 |
| BDDS_5 -- BDDS_6 | 0.2318107648 | 0.2272566965 | 0.080093964 | 0.378817029 |
| BIDQ_1 -- BDDS_7 | 0.0932730295 | 0.0847198120 | 0.000000000 | 0.186695037 |
| BIDQ_2 -- BDDS_7 | 0.0338679626 | 0.0514074639 | -0.002129899 | 0.148818184 |
| BIDQ_3 -- BDDS_7 | -0.0102606512 | -0.0260270723 | -0.134348817 | 0.053685914 |
| BIDQ_4 -- BDDS_7 | 0.0000000000 | 0.0068394470 | -0.093554708 | 0.113777383 |
| BIDQ_5 -- BDDS_7 | 0.0000000000 | -0.0184223214 | -0.139338696 | 0.078444394 |
| BIDQ_6 -- BDDS_7 | -0.1472570834 | -0.1441159792 | -0.276676811 | -0.009946495 |
| BIDQ_7 -- BDDS_7 | 0.0174753715 | 0.0340760354 | -0.037570415 | 0.133678880 |
| BDDS_1 -- BDDS_7 | 0.0193654289 | 0.0336609977 | -0.113928418 | 0.189075257 |
| BDDS_2 -- BDDS_7 | 0.1602132590 | 0.1569104103 | 0.000000000 | 0.313158825 |
| BDDS_3 -- BDDS_7 | 0.0629778861 | 0.0653170412 | -0.034481230 | 0.202348011 |
| BDDS_4 -- BDDS_7 | -0.0628481858 | -0.0809343130 | -0.243987982 | 0.036860653 |
| BDDS_5 -- BDDS_7 | 0.1463357328 | 0.1489178524 | 0.002904462 | 0.306250372 |
| BDDS_6 -- BDDS_7 | 0.3070152382 | 0.3157685016 | 0.177009398 | 0.440086183 |
| BIDQ_1 -- BDDS_8 | 0.0111990822 | 0.0335540928 | -0.040584308 | 0.137449360 |
| BIDQ_2 -- BDDS_8 | -0.0175216729 | -0.0317730943 | -0.144394955 | 0.043111391 |
| BIDQ_3 -- BDDS_8 | 0.0943542913 | 0.1027509941 | 0.000000000 | 0.227498433 |
| BIDQ_4 -- BDDS_8 | 0.0203347388 | 0.0215029196 | -0.083104930 | 0.135565193 |
| BIDQ_5 -- BDDS_8 | 0.0895515245 | 0.0951513301 | 0.000000000 | 0.227090654 |
| BIDQ_6 -- BDDS_8 | -0.0731177030 | -0.0764261191 | -0.221386568 | 0.011227849 |
| BIDQ_7 -- BDDS_8 | -0.0511643445 | -0.0641351633 | -0.183334685 | 0.007033425 |
| BDDS_1 -- BDDS_8 | 0.2673371917 | 0.2807778607 | 0.114943301 | 0.433413663 |
| BDDS_2 -- BDDS_8 | 0.0000000000 | -0.0287128048 | -0.197169226 | 0.110479187 |
| BDDS_3 -- BDDS_8 | -0.0827362380 | -0.0946295602 | -0.249008394 | 0.017851817 |
| BDDS_4 -- BDDS_8 | 0.0847875173 | 0.0909106192 | -0.020064401 | 0.247776867 |
| BDDS_5 -- BDDS_8 | -0.0531426551 | -0.0630528822 | -0.240148567 | 0.065612115 |
| BDDS_6 -- BDDS_8 | 0.0982602781 | 0.0987405279 | -0.019446147 | 0.260849234 |
| BDDS_7 -- BDDS_8 | 0.2391244259 | 0.2445980378 | 0.077452266 | 0.398772658 |
| BIDQ_1 -- BDDS_9 | -0.0786111682 | -0.0801674454 | -0.193881594 | 0.000000000 |
| BIDQ_2 -- BDDS_9 | -0.0115052955 | -0.0257268331 | -0.133662543 | 0.062705198 |
| BIDQ_3 -- BDDS_9 | -0.0191998774 | -0.0314675475 | -0.141180244 | 0.049579818 |
| BIDQ_4 -- BDDS_9 | 0.0075175321 | 0.0143093928 | -0.082987869 | 0.132816526 |
| BIDQ_5 -- BDDS_9 | 0.0000000000 | 0.0121567880 | -0.102190986 | 0.131107665 |
| BIDQ_6 -- BDDS_9 | 0.0990864069 | 0.0926649228 | -0.004565157 | 0.229118336 |
| BIDQ_7 -- BDDS_9 | 0.0041087752 | 0.0154708020 | -0.075920596 | 0.114824214 |
| BDDS_1 -- BDDS_9 | 0.0000000000 | -0.0088882275 | -0.171760885 | 0.129582832 |
| BDDS_2 -- BDDS_9 | 0.0715870143 | 0.0872136853 | -0.034818371 | 0.254406080 |
| BDDS_3 -- BDDS_9 | 0.0456320136 | 0.0544137623 | -0.072895980 | 0.201246090 |
| BDDS_4 -- BDDS_9 | 0.0409467089 | 0.0414365875 | -0.091577099 | 0.188999225 |
| BDDS_5 -- BDDS_9 | 0.1723848607 | 0.1736596839 | 0.004752347 | 0.330885246 |
| BDDS_6 -- BDDS_9 | -0.0710477460 | -0.0798713578 | -0.250178935 | 0.046017887 |
| BDDS_7 -- BDDS_9 | 0.1311118371 | 0.1302713576 | -0.001870642 | 0.301909625 |
| BDDS_8 -- BDDS_9 | 0.3449324708 | 0.3484908763 | 0.210029518 | 0.479082444 |
| BIDQ_1 -- BDDS_10 | 0.0000000000 | -0.0029290351 | -0.092988415 | 0.087761082 |
| BIDQ_2 -- BDDS_10 | 0.0000000000 | 0.0044447175 | -0.080186094 | 0.100115800 |
| BIDQ_3 -- BDDS_10 | -0.0001182197 | -0.0251678702 | -0.144901597 | 0.060173590 |
| BIDQ_4 -- BDDS_10 | 0.1088141186 | 0.1182115558 | 0.000000000 | 0.262019862 |
| BIDQ_5 -- BDDS_10 | -0.0530059697 | -0.0520575380 | -0.202798626 | 0.051174794 |
| BIDQ_6 -- BDDS_10 | 0.0000000000 | -0.0175632945 | -0.152140956 | 0.098245949 |
| BIDQ_7 -- BDDS_10 | 0.0207708842 | 0.0484114843 | -0.038576823 | 0.178577740 |
| BDDS_1 -- BDDS_10 | 0.0000000000 | -0.0189828778 | -0.187591642 | 0.124270287 |
| BDDS_2 -- BDDS_10 | 0.1423383997 | 0.1460491539 | 0.000000000 | 0.303919909 |
| BDDS_3 -- BDDS_10 | 0.0000000000 | 0.0121152727 | -0.119635004 | 0.153186726 |
| BDDS_4 -- BDDS_10 | 0.1136192214 | 0.1060304552 | -0.024477748 | 0.270715503 |
| BDDS_5 -- BDDS_10 | 0.0733063870 | 0.0817387851 | -0.072169036 | 0.245604190 |
| BDDS_6 -- BDDS_10 | 0.0345612986 | 0.0424414502 | -0.097106505 | 0.203071849 |
| BDDS_7 -- BDDS_10 | -0.2160431704 | -0.2317371933 | -0.441516930 | -0.040208872 |
| BDDS_8 -- BDDS_10 | 0.1211622606 | 0.1410903526 | 0.000000000 | 0.334756408 |
| BDDS_9 -- BDDS_10 | 0.1183016408 | 0.1189736703 | -0.019366689 | 0.298476379 |
| BIDQ_1 -- BDDS_11 | -0.0537987577 | -0.0557531611 | -0.169905210 | 0.022413364 |
| BIDQ_2 -- BDDS_11 | -0.0369413296 | -0.0457481062 | -0.163180891 | 0.025198976 |
| BIDQ_3 -- BDDS_11 | 0.0000000000 | 0.0320736282 | -0.057899772 | 0.152236904 |
| BIDQ_4 -- BDDS_11 | -0.0997053289 | -0.1117347118 | -0.262039310 | 0.000000000 |
| BIDQ_5 -- BDDS_11 | -0.0054022984 | -0.0159836326 | -0.155609601 | 0.116371425 |
| BIDQ_6 -- BDDS_11 | 0.1734146879 | 0.1916632859 | 0.056212646 | 0.330446378 |
| BIDQ_7 -- BDDS_11 | -0.0204629940 | -0.0446381929 | -0.179902019 | 0.045702682 |
| BDDS_1 -- BDDS_11 | 0.0681488881 | 0.0749482890 | -0.054176112 | 0.245717047 |
| BDDS_2 -- BDDS_11 | 0.0176342634 | 0.0290800869 | -0.119443167 | 0.191219975 |
| BDDS_3 -- BDDS_11 | 0.0000000000 | -0.0182412793 | -0.164512405 | 0.111109360 |
| BDDS_4 -- BDDS_11 | 0.0150626755 | 0.0216204162 | -0.135786564 | 0.174269336 |
| BDDS_5 -- BDDS_11 | 0.1236441656 | 0.1172705424 | -0.014172495 | 0.277549852 |
| BDDS_6 -- BDDS_11 | 0.0481033362 | 0.0529132022 | -0.097022554 | 0.214229464 |
| BDDS_7 -- BDDS_11 | 0.1751660221 | 0.1824718320 | 0.000000000 | 0.381160821 |
| BDDS_8 -- BDDS_11 | 0.0000000000 | -0.0138890912 | -0.216925503 | 0.152302489 |
| BDDS_9 -- BDDS_11 | 0.0000000000 | -0.0055907933 | -0.188579269 | 0.159564132 |
| BDDS_10 -- BDDS_11 | 0.5636192559 | 0.5653442070 | 0.435915452 | 0.679010327 |

## Supplementary Table S5. Bridge Centrality by Gender

Bridge centrality estimates derived from gender-specific symptom networks, including bridge strength and bridge expected influence indices for male and female participants.

| Node | Male_BridgeStrength | Female_BridgeStrength | Male_BridgeEI_1step | Female_BridgeEI_1step | Male_BridgeEI_2step | Female_BridgeEI_2step |
| --- | --- | --- | --- | --- | --- | --- |
| BIDQ_1 | 0.6338260 | 0.5785346 | 0.12196367 | 0.16174270 | 0.231868399 | 0.24396260 |
| BIDQ_2 | 0.4082913 | 0.2853318 | 0.10611752 | 0.10409640 | 0.265174564 | 0.32936533 |
| BIDQ_3 | 0.4102208 | 0.4998491 | 0.01427182 | 0.15369140 | 0.042656748 | 0.29697296 |
| BIDQ_4 | 0.8849838 | 0.3427325 | 0.01079314 | 0.06611666 | 0.073241251 | 0.20219912 |
| BIDQ_5 | 0.6308097 | 0.5135041 | -0.02780195 | 0.03521025 | -0.007157779 | 0.18032176 |
| BIDQ_6 | 0.7377710 | 0.4955016 | 0.07751338 | 0.04672467 | 0.264567371 | 0.15374648 |
| BIDQ_7 | 0.5274837 | 0.4047187 | 0.06231303 | 0.15272828 | 0.124540187 | 0.33016660 |
| BDDS_1 | 0.3970799 | 0.1433856 | 0.05179943 | 0.02148507 | 0.056436440 | 0.20787805 |
| BDDS_2 | 0.1801170 | 0.2533035 | 0.05189891 | 0.25330351 | 0.146971146 | 0.42242222 |
| BDDS_3 | 0.4041158 | 0.3284153 | -0.10143610 | 0.20584648 | -0.138771427 | 0.29747138 |
| BDDS_4 | 0.4538591 | 0.3220533 | -0.04788365 | 0.12905943 | -0.079921191 | 0.35822523 |
| BDDS_5 | 0.4089599 | 0.2066480 | 0.06464176 | 0.08829415 | 0.224749403 | 0.16803149 |
| BDDS_6 | 0.4609475 | 0.1491768 | 0.11660581 | 0.02139223 | 0.182013051 | 0.02849649 |
| BDDS_7 | 0.4260880 | 0.3223056 | 0.04119068 | -0.05525122 | 0.074744544 | 0.01126438 |
| BDDS_8 | 0.4156793 | 0.2099152 | -0.10221952 | 0.20991523 | 0.002327407 | 0.27622445 |
| BDDS_9 | 0.2894724 | 0.3641457 | 0.19365202 | -0.22320936 | 0.308664530 | -0.19205088 |
| BDDS_10 | 0.2928280 | 0.3131512 | 0.13785749 | 0.12246136 | 0.196414415 | 0.09082373 |
| BDDS_11 | 0.5042395 | 0.5076722 | -0.04093624 | -0.05298653 | 0.021262424 | 0.06794831 |

## Supplementary Table S6. NCT Significant Edgewise Differences

Edges showing statistically significant differences between male and female participants based on the Network Comparison Test.

| Var1 | Var2 | p-value | Test statistic E |
| --- | --- | --- | --- |
| BIDQ_3 | BIDQ_4 | 0.002997003 | 0.19090484 |
| BIDQ_4 | BIDQ_5 | 0.002997003 | 0.22176007 |
| BIDQ_3 | BDDS_1 | 0.029970030 | 0.02717437 |
| BIDQ_4 | BDDS_1 | 0.015984016 | 0.04563768 |
| BIDQ_7 | BDDS_2 | 0.041958042 | 0.05834102 |
| BDDS_1 | BDDS_2 | 0.043956044 | 0.13915221 |
| BIDQ_1 | BDDS_3 | 0.000999001 | 0.08674258 |
| BIDQ_5 | BDDS_4 | 0.006993007 | 0.05335358 |
| BIDQ_7 | BDDS_4 | 0.006993007 | 0.07916962 |
| BDDS_3 | BDDS_4 | 0.012987013 | 0.12006917 |
| BIDQ_5 | BDDS_6 | 0.012987013 | 0.07360579 |
| BDDS_2 | BDDS_7 | 0.039960040 | 0.13489792 |
| BIDQ_7 | BDDS_8 | 0.019980020 | 0.01299953 |
| BDDS_6 | BDDS_8 | 0.020979021 | 0.14725698 |
| BIDQ_2 | BDDS_9 | 0.035964036 | 0.00690233 |
| BIDQ_3 | BDDS_9 | 0.009990010 | 0.03099051 |
| BDDS_3 | BDDS_10 | 0.003996004 | 0.05547144 |
| BDDS_9 | BDDS_10 | 0.001998002 | 0.22977708 |
| BDDS_1 | BDDS_11 | 0.020979021 | 0.13778581 |

## Supplementary Table S7. NCT Centrality Invariance Results

Permutation-based p-values for node strength invariance between male and female participants obtained from the Network Comparison Test.

| Node | Strength_p |
| --- | --- |
| BIDQ_1 | 0.72027972 |
| BIDQ_2 | 0.37162837 |
| BIDQ_3 | 0.18881119 |
| BIDQ_4 | 0.33466533 |
| BIDQ_5 | 0.32967033 |
| BIDQ_6 | 0.75224775 |
| BIDQ_7 | 0.36263736 |
| BDDS_1 | 0.35364635 |
| BDDS_2 | 0.63036963 |
| BDDS_3 | 0.99400599 |
| BDDS_4 | 0.25974026 |
| BDDS_5 | 0.02297702 |
| BDDS_6 | 0.20179820 |
| BDDS_7 | 0.52047952 |
| BDDS_8 | 0.07192807 |
| BDDS_9 | 0.74025974 |
| BDDS_10 | 0.88911089 |
| BDDS_11 | 0.19380619 |

## Supplementary Figure S1. Bootstrap Edge-Weight Confidence Intervals

Bootstrap confidence intervals for estimated network edge weights.


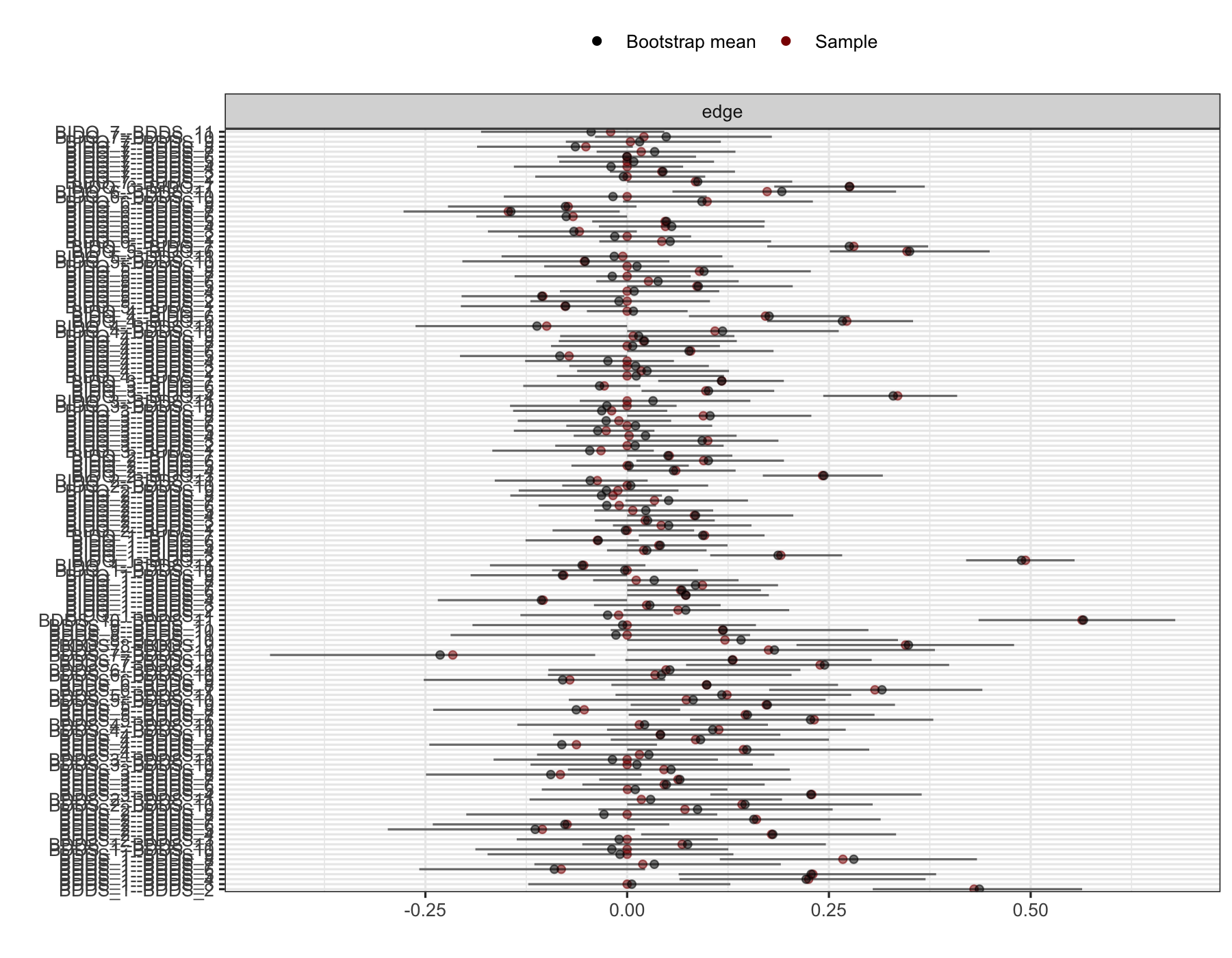


## Supplementary Figure S2. Bootstrap Edge-Weight Difference Plot

Bootstrap difference test comparing estimated edge weights across the network.


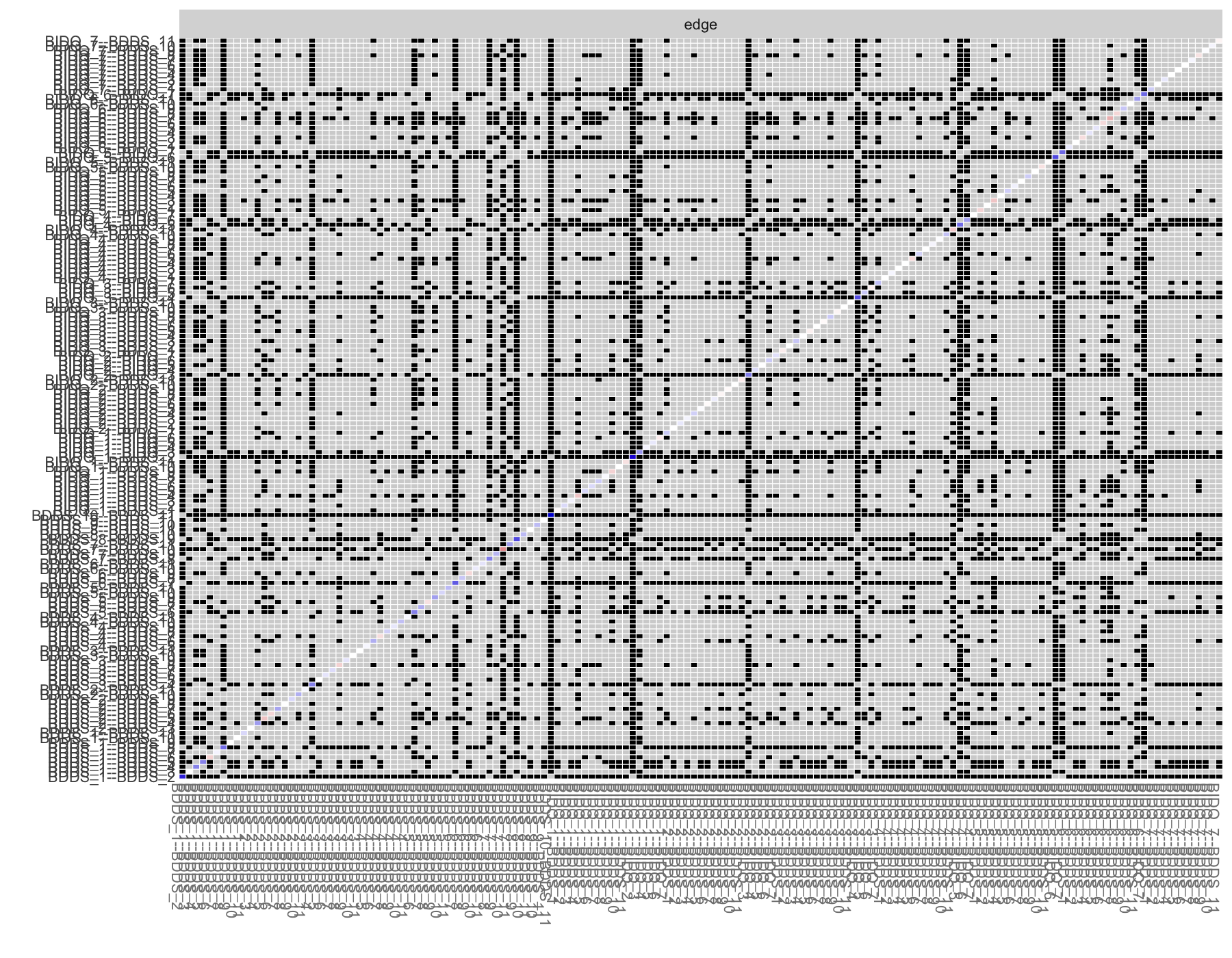


# Sensitivity Analysis

To examine the robustness of the findings, a sensitivity analysis was conducted by estimating an alternative network that additionally included BDDS_12, the BDDS-5 exclusion item related to weight- and eating-disorder concerns.

## Supplementary Table S8. Centrality Comparison Across Network Specifications

Comparison of node-wise centrality estimates between the primary symptom network and the alternative network that additionally included BDDS_12, the BDDS-5 exclusion item related to weight- and eating-disorder concerns.

| Node | Strength_noD | ExpectedInfluence_1step_noD | Predictability_R2_noD | Strength_withD | ExpectedInfluence_1step_withD | Predictability_R2_withD |
| --- | --- | --- | --- | --- | --- | --- |
| BDDS_1 | 1.5688146 | 1.1656359 | 0.38751833 | 1.4324821 | 1.1418174 | 0.35831168 |
| BDDS_10 | 1.5656608 | 1.0273261 | 0.44783633 | 1.5758807 | 0.6985569 | 0.39717636 |
| BDDS_11 | 1.4011040 | 0.9684826 | 0.41585443 | 1.4253114 | 0.6762366 | 0.35604975 |
| BDDS_12 |  |  |  | 0.9869830 | -0.5144483 | 0.13573357 |
| BDDS_2 | 1.3027305 | 0.9437736 | 0.29080515 | 1.2934949 | 0.8241601 | 0.27844042 |
| BDDS_3 | 0.8195385 | 0.3267332 | 0.09468477 | 0.7378412 | 0.3183653 | 0.08178966 |
| BDDS_4 | 1.3463345 | 1.0125443 | 0.20186603 | 1.2489586 | 1.0056299 | 0.18424654 |
| BDDS_5 | 1.4623673 | 0.9500070 | 0.21342032 | 1.2471429 | 0.8762157 | 0.16815316 |
| BDDS_6 | 1.3849817 | 0.7770941 | 0.21712507 | 1.2850888 | 0.6090573 | 0.19952835 |
| BDDS_7 | 1.8223353 | 0.9495171 | 0.33330273 | 1.6393508 | 0.9326345 | 0.29612056 |
| BDDS_8 | 1.6487264 | 1.0933612 | 0.31455393 | 1.5371985 | 1.2678935 | 0.29290916 |
| BDDS_9 | 1.2159733 | 0.8552452 | 0.21038417 | 1.2448086 | 1.0160523 | 0.20352218 |
| BIDQ_1 | 1.4538718 | 0.8860858 | 0.33543035 | 1.3442138 | 0.8874049 | 0.32190463 |
| BIDQ_2 | 1.2074647 | 1.0558812 | 0.32990659 | 1.1470718 | 1.0815030 | 0.32260373 |
| BIDQ_3 | 1.2955057 | 1.0629456 | 0.25241957 | 1.2443687 | 1.0032697 | 0.24144712 |
| BIDQ_4 | 1.2637738 | 0.9204288 | 0.25375824 | 1.1776441 | 0.9204358 | 0.23813540 |
| BIDQ_5 | 1.4787219 | 0.9989974 | 0.32008046 | 1.3741251 | 0.9688744 | 0.29897560 |
| BIDQ_6 | 1.7105399 | 0.8869710 | 0.31808325 | 1.5272051 | 0.8582136 | 0.28085435 |
| BIDQ_7 | 1.0637904 | 0.9205357 | 0.19346818 | 0.9684790 | 0.9121707 | 0.18493865 |

## Supplementary Table S9. Bridge Centrality Comparison Across Network Specifications

Comparison of bridge centrality estimates between the primary symptom network and the alternative network that additionally included BDDS_12, the BDDS-5 exclusion item related to weight- and eating-disorder concerns.

| Node | BridgeStrength_noD | BridgeEI_1step_noD | BridgeStrength_withD | BridgeEI_1step_withD |
| --- | --- | --- | --- | --- |
| BDDS_1 | 0.2475319 | 0.007657083 | 0.1717050 | 0.01214477 |
| BDDS_10 | 0.1827092 | 0.076460813 | 0.1248896 | 0.05281129 |
| BDDS_11 | 0.3897254 | -0.042896021 | 0.3177003 | -0.03539896 |
| BDDS_12 |  |  | 0.1028413 | -0.05814218 |
| BDDS_2 | 0.1226899 | 0.122689900 | 0.1021901 | 0.10219010 |
| BDDS_3 | 0.3530902 | 0.025757380 | 0.3257657 | 0.02201495 |
| BDDS_4 | 0.2372493 | 0.029155452 | 0.2084660 | 0.03624396 |
| BDDS_5 | 0.3109280 | 0.115025242 | 0.2423377 | 0.10698026 |
| BDDS_6 | 0.2481884 | 0.094484762 | 0.1980327 | 0.08718272 |
| BDDS_7 | 0.3021341 | -0.012901371 | 0.2552172 | -0.01561909 |
| BDDS_8 | 0.3572434 | 0.073635916 | 0.2235581 | 0.08966220 |
| BDDS_9 | 0.2200291 | 0.001396373 | 0.1784404 | 0.01200848 |
| BIDQ_1 | 0.5774177 | 0.083352823 | 0.5029932 | 0.07916864 |
| BIDQ_2 | 0.2645487 | 0.112965151 | 0.2100470 | 0.14447819 |
| BIDQ_3 | 0.2846028 | 0.108706403 | 0.2708037 | 0.05280530 |
| BIDQ_4 | 0.4044196 | 0.061074610 | 0.3154373 | 0.05822901 |
| BIDQ_5 | 0.4423320 | -0.037392499 | 0.3518797 | -0.05337100 |
| BIDQ_6 | 0.7565702 | 0.063386023 | 0.6597980 | 0.04689133 |
| BIDQ_7 | 0.2416277 | 0.098373019 | 0.1401853 | 0.08387702 |

## Supplementary Table S10. Network Comparison Test Summary Across Network Specifications

Comparison of key Network Comparison Test statistics between the primary symptom network and the alternative network that additionally included BDDS_12, the BDDS-5 exclusion item related to weight- and eating-disorder concerns.

| Metric | NoD | WithD |
| --- | --- | --- |
| GlobalStrength_Male | 7.85623504 | 8.26284426 |
| GlobalStrength_Female | 8.13414641 | 8.55648874 |
| GlobalStrength_Difference | 0.27791136 | 0.29364448 |
| GlobalStrength_p | 0.13986014 | 0.13486513 |
| NetworkStructure_M | 0.22977708 | 0.23309439 |
| NetworkStructure_p | 0.04095904 | 0.02797203 |

## Supplementary Figure S4. Sensitivity Analysis Network

Symptom network estimated in the sensitivity analysis, in which BDDS_12, the BDDS-5 exclusion item related to weight- and eating-disorder concerns, was additionally included.


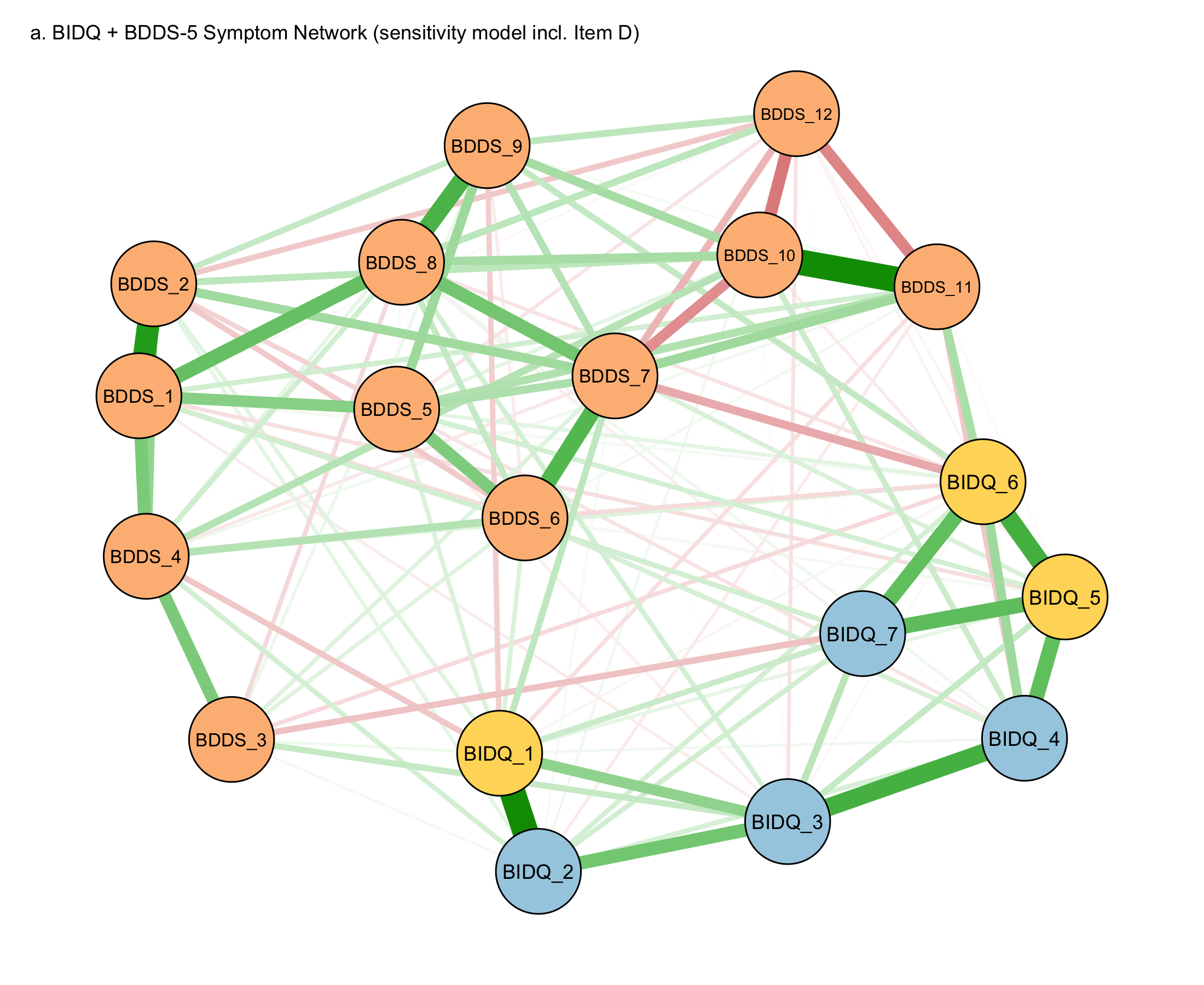

Supplement: Supplementary file 1 — Supplementary Materials: brb371538‐sup‐0001‐SuppMat.docx [file BRB3-16-e71538-s001.docx]
